# Microbially catalyzed conjugation of GABA and tyramine to bile acids

**DOI:** 10.1128/jb.00426-23

**Published:** 2024-01-04

**Authors:** Michael W. Mullowney, Aretha Fiebig, Matthew K. Schnizlein, Mary McMillin, Amber R. Rose, Jason Koval, David Rubin, Sushila Dalal, Mitchell L. Sogin, Eugene B. Chang, Ashley M. Sidebottom, Sean Crosson

**Affiliations:** 1 Duchossois Family Institute, University of Chicago, Chicago, Illinois, USA; 2 Department of Microbiology and Molecular Genetics, Michigan State University, East Lansing, Michigan, USA; 3 Department of Medicine, University of Chicago, Chicago, Illinois, USA; 4 Marine Biological Laboratory, Woods Hole, Massachusetts, USA; Geisel School of Medicine at Dartmouth, Hanover, New Hampshire, USA

**Keywords:** bile acid, GABA, *Bacteroides*, neurotransmitter, gut microbiome, colitis, metabolomics, mass spectrometry, bifidobacteria

## Abstract

Bile acids (BAs) are cholesterol-derived molecules that aid in digestion and nutrient absorption, regulate host metabolic processes, and influence physiology of the gut microbiota. Both the host and its microbiome contribute to enzymatic modifications that shape the chemical diversity of BAs in the gut. Several bacterial species have been reported to conjugate standard amino acids to BAs, but it was not known if bacteria conjugate BAs to other amine classes. Here, we show that *Bacteroides fragilis* strain P207, isolated from a bacterial bloom in the J-pouch of a patient with ulcerative colitis pouchitis, conjugates standard amino acids and the neuroactive amines γ-aminobutyric acid (GABA) and tyramine to deoxycholic acid. We extended this analysis to other human gut isolates and identified species that are competent to conjugate GABA and tyramine to primary and secondary BAs, and further identified diverse BA-GABA and BA-tyramine amides in human stool. A longitudinal metabolomic analysis of J-pouch contents of the patient from whom *B. fragilis* P207 was isolated revealed highly reduced levels of secondary bile acids and a shifting BA amide profile before, during, and after onset of pouchitis, including temporal changes in several BA-GABA amides. Treatment of pouchitis with ciprofloxacin was associated with a marked reduction of nearly all BA amides in the J-pouch. Our study expands the known repertoire of conjugated bile acids produced by bacteria to include BA conjugates to GABA and tyramine and demonstrates that these molecules are present in the human gut.

**IMPORTANCE:**

BAs are modified in multiple ways by host enzymes and the microbiota to produce a chemically diverse set of molecules that assist in the digestive process and impact many physiological functions. This study reports the discovery of bacterial species that conjugate the neuroactive amines, GABA and tyramine, to primary and secondary BAs. We further present evidence that BA-GABA and BA-tyramine conjugates are present in the human gut, and document a shifting BA-GABA profile in a human pouchitis patient before, during, and after inflammation and antibiotic treatment. GABA and tyramine are common metabolic products of the gut microbiota and potent neuroactive molecules. GABA- and tyramine-conjugated BAs may influence receptor-mediated regulatory mechanisms of humans and their gut microbes, and absorption of these molecules and their entry into enterohepatic circulation may impact host physiology at distal tissue sites. This study defines new conjugated bile acids in the human gut.

## INTRODUCTION

Primary bile acids (PBAs) are produced from cholesterol in the liver through a multi-step enzymatic process (1). In the hepatocyte, PBAs are enzymatically conjugated to the amine group of glycine (Gly) or taurine to form an amide bond (2, 3) and are eventually secreted into bile and released into the intestinal lumen. Once in the gut, members of the microbiota deconjugate glycine or taurine from PBAs and chemically modify the sterol core, yielding secondary bile acids (SBAs) of various chemical forms (4, 5). This chemically diverse ensemble of bile acids (BAs) in the human gut are eventually resorbed in the intestine and returned to the liver through enterohepatic circulation, where they can be re-conjugated or repaired in the hepatocyte (6). Together, PBAs and SBAs in their various chemical forms play important roles in digestion and absorption of dietary fats and regulate a range of host (1) and microbial (7) metabolic processes.

Recent studies have shown that the majority of standard protein-encoding amino acids can be conjugated to both PBAs and SBAs by bacteria that reside in the gut including species of the genera *Bacteroides*, *Lactobacillus*, *Bifidobacterium*, *Enterocloster*, *Ruminococcus*, and *Clostridium* (8 – 10). This discovery has expanded the known chemical repertoire of BAs present in humans, though the impact of these microbially conjugated BAs on host and microbial physiology is not known. Certainly, the potent antibacterial properties of BAs have been recognized for over a century (11, 12), and it is long established that conjugated forms of BAs are less inhibitory to growth of intestinal microflora than unconjugated free acids *in vitro* (13 – 15). Gut bacteria may also encode protein receptors that cue specific regulatory responses to BAs including virulence gene expression (16, 17) and spore germination (18). The VtrA-VtrB-VtrC regulatory system of *Vibrio parahaemolyticus* is a notable example of the specificity of microbial responses to BAs. Though both conjugated and unconjugated bile acids bind the VtrA-VtrC receptor complex at the same site and with similar affinities (19), only specific BA species cue the transcription of the type III secretion regulator, *vtrB* (16, 17, 19). Beyond the effect of BAs on the microbiota, the impact of these molecules on mammalian physiology is well established and dependent on the chemical form of the molecule (20). For example, Takeda G protein-coupled receptor 5 (TGR5) and farnesoid X receptor can function as bile acid receptors, and signaling through these receptors is influenced by the chemical identity of the amino acid that is conjugated to the sterol core (10, 21).

In ulcerative colitis (UC) patients who have undergone ileal pouch anal anastomosis (IPAA), a dysbiosis-induced deficiency of SBAs including deoxycholic acid (DCA) and lithocholic acid (LCA) is proposed to cue an inflammatory state that can lead to pouchitis (22). It is not known if microbial bile acid conjugation reactions directly modulate DCA and LCA levels in these patients, but genera with the capacity to conjugate amino acids to BAs (9), including *Bacteroides* spp., often dominate the inflamed ileoanal pouch (or J-pouch) (23). We have previously isolated *Bacteroides fragilis* strains that constitute over 50% of the bacterial population of the J-pouch before the emergence of inflammation (23). We aimed to test the ability of a predominant *B. fragilis* strain (P207) to conjugate amino acids and other amines present in the gut to primary and secondary bile acids. We further aimed to define the temporal bile acid profile of the J-pouch of the human patient from whom this particular *B. fragilis* strain was isolated.

Here, we report that *B. fragilis* strain P207 conjugates Gly, alanine (Ala), phenylalanine (Phe), γ-aminobutyric acid (GABA), and tyramine to DCA *in vitro*; conjugation to CA was limited to glycine. *B. fragilis* P207 deconjugates glycodeoxycholic acid (GDCA) to produce DCA and can subsequently produce Ala-, Phe-, GABA-, and tyramine-DCA conjugates from the deconjugated bile acid. Thus, *B. fragilis* P207 produces a chemically diverse pool of conjugated bile acids *in vitro*, including the novel GABA-DCA and tyramine-DCA products. A time-series metabolomic analysis of stool from pouchitis patient 207 before and after the onset of inflammation revealed severely diminished secondary bile acids across all time points and the presence of multiple bile acid-amine conjugates, the levels of which were strongly reduced following antibiotic treatment. Among the BA amides detected in pouch samples or in samples from healthy human donors, were diverse GABA and tyramine conjugates. GABA- and tyramine-BA amide synthesis was not limited to *B. fragilis* P207, as we identified other classes of gut anaerobes that conjugate these neuroactive amines to PBAs and SBAs. Our results expand the known set of microbially catalyzed bile conjugation reactions and have identified novel bile acid conjugates to GABA and tyramine in the human gut. Both GABA (24) and tyramine (25) are common products of microbial metabolism and are potent neuromodulators. Flux of these amines in the gut due to microbe-catalyzed conjugation to bile acids may impact host physiology.

## RESULTS

### Chemical transformation of primary and secondary bile acids by *B. fragilis* P207

**Fig 1:**
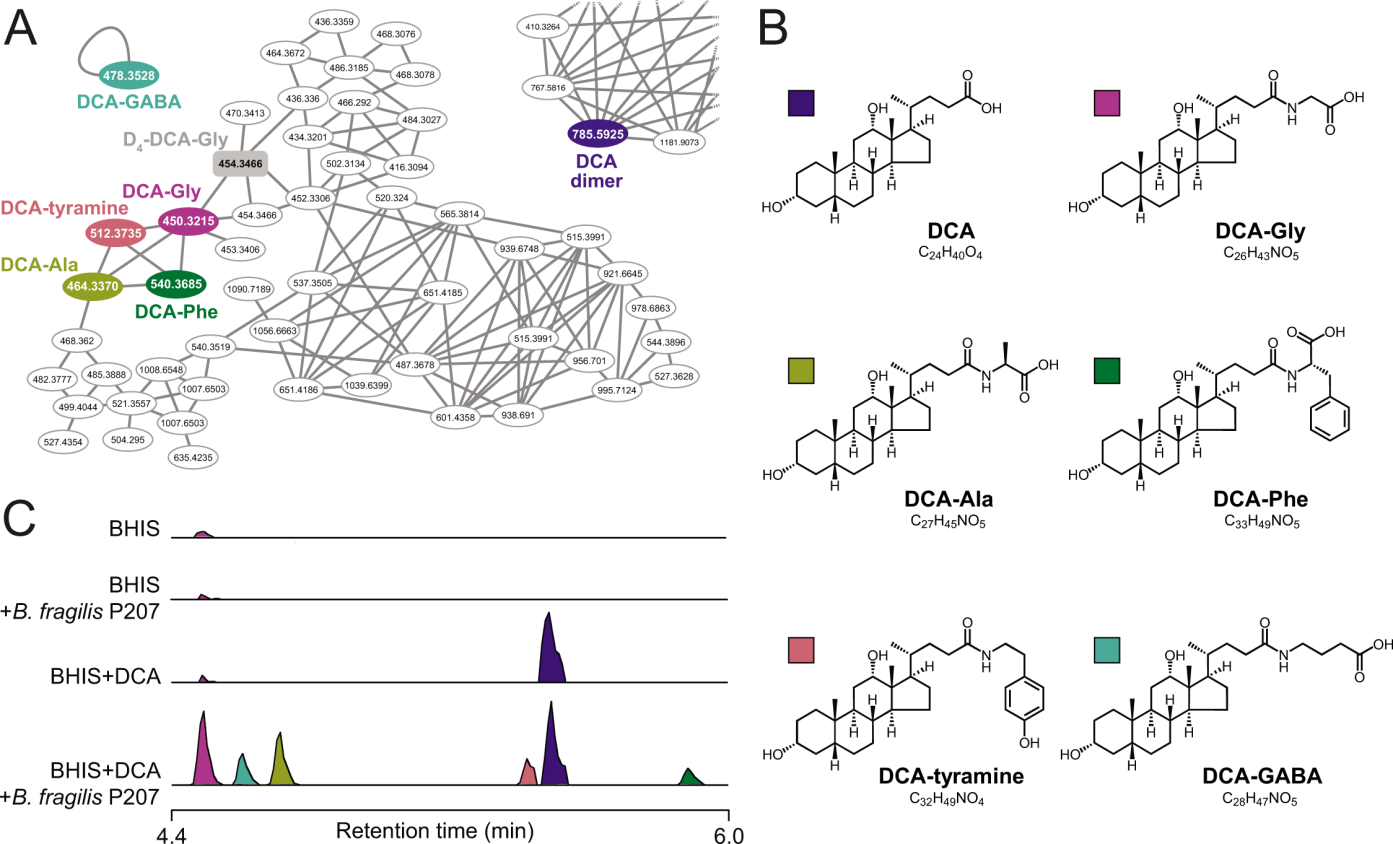
Identification of bile acid conjugates produced by pouchitis patient isolate, *B. fragilis* P207 by UHPLC-MS^2^ (**A**) Molecular family containing nodes for deoxycholic acid (DCA)-amine conjugates. Colored nodes represent features only observed in *B. fragilis* P207 cultures with 0.01% (wt/vol) DCA present. The gray-shaded rectangular node is the D_4_-DCA-glycine (Gly) internal standard. (**B**) Structures of the five DCA-amine conjugates with color key for panels A–C. (**C**) Stacked selected ion chromatograms for the five DCA-amino acid conjugates detected in BHIS media (BHIS), *B. fragilis* strain P207 culture extract (BHIS + *B. fragilis* 207), BHIS media with 0.01% DCA (BHIS + DCA), and *B. fragilis* strain P207 culture extract with DCA (BHIS + *B. fragilis* P207 + DCA).

**Fig 2:**
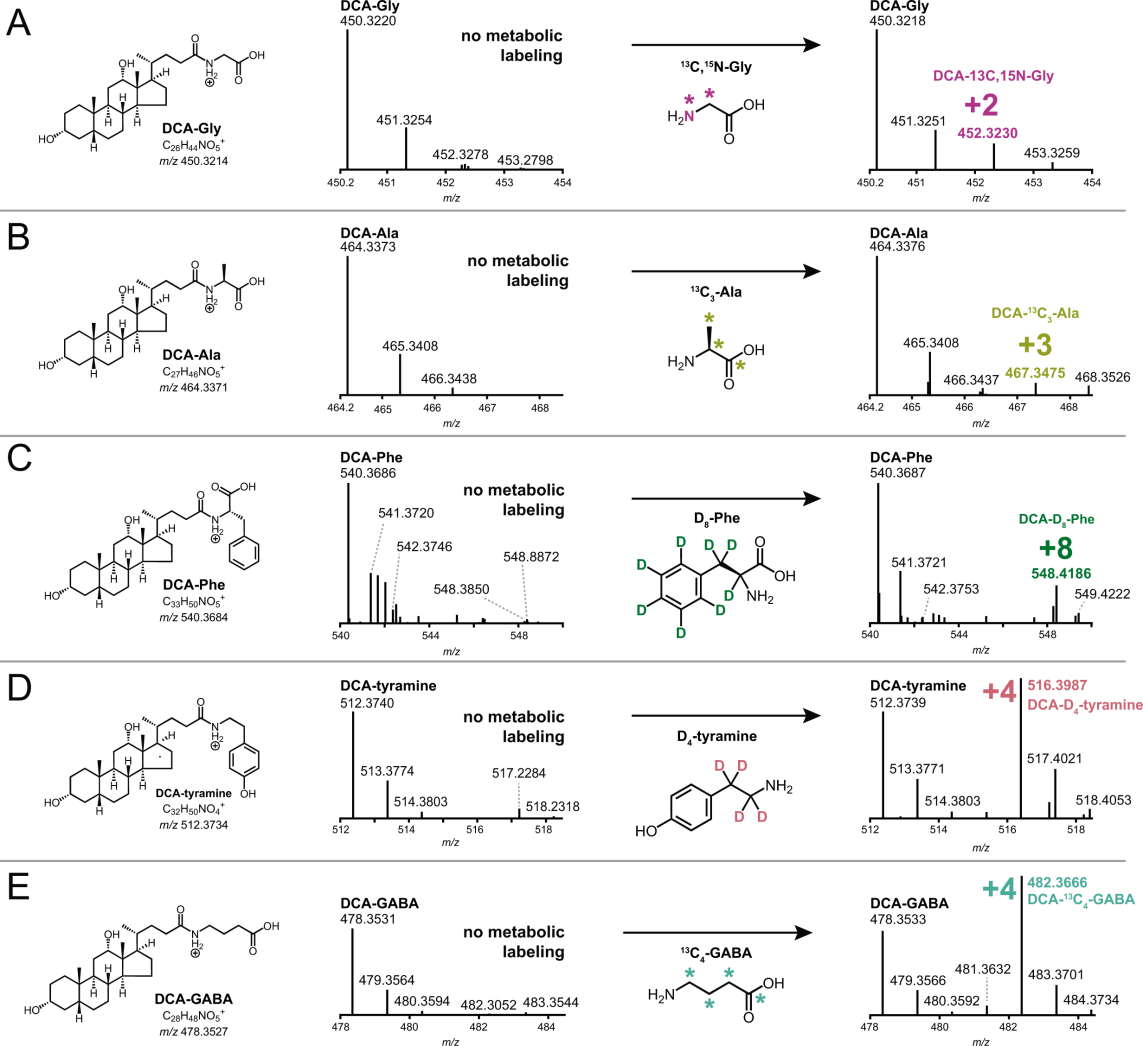
Amine feeding experiments to test bile acid conjugation by *B. fragilis* strain P207. Mass spectrometry analysis was performed to assess production of bile acid conjugates with five different amines: glycine (**A**), alanine (**B**), phenylalanine (**C**), tyramine (**D**), and GABA (**E**). *B. fragilis* P207 was fed isotopically labeled versions of these amines. Observed mass to charge (*m*/*z*) shifts, displayed in each panel, are consistent with the expected molecular weights for the respective bile acid-heavy amino acid/amine conjugates presented in Fig. 1.

**Fig 3:**
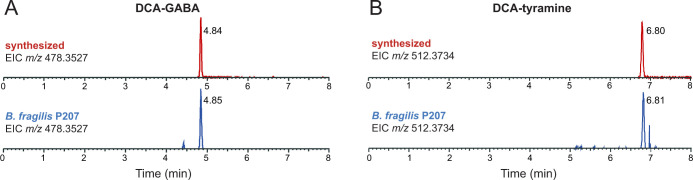
Extracted ion chromatograms (EICs) of deoxycholate conjugates produced by *B. fragilis* P207 *in vitro* compared to synthetic standards. (**A**) EIC of chemically synthesized deoxycholic acid (DCA)-GABA (*m*/*z* 478.3527) (top, retention time = 4.84 min) and the EIC of DCA-GABA identified in *B. fragilis* P207 *in vitro* cultures (bottom, retention time = 4.85 min). (**B**) EIC of chemically synthesized DCA-tyramine (*m*/*z* 512.3734) (top, retention time = 6.80 min) and the EIC of DCA-tyramine identified in *B. fragilis* P207 *in vitro* cultures (bottom, retention time = 6.81 min). After identifying bile acid conjugates as key metabolites (Fig. 1), we refined the chromatography method to effectively separate the complete array of possible conjugate isomers, considering both DCA and cholic acid isomers as additional potential substrates. This accounts for the differences in retention times from Fig. 1.

*B. fragilis* P207 was incubated in supplemented brain-heart infusion (BHIS) broth with and without DCA (0.01% wt/vol), and samples were analyzed by ultra-high-performance liquid chromatography tandem high-resolution mass spectrometry (UHPLC-MS^2^). Fragmentation-based networking of the mass spectrometry data identified derivatives of DCA from a set of candidate conjugates presented in Table S1. This metabolomic approach provided evidence for five amide-linked conjugates of DCA, which were only present in the spent media of cultures that contained both *B. fragilis* P207 and DCA. Fragmentation patterns consistent with DCA-alanine, DCA-glycine, DCA-phenylalanine, DCA-tyramine, and DCA-GABA were identified (Fig. 1). We aimed to validate the synthesis of bile acid-amine conjugates by *B. fragilis* P207 and to investigate if this strain could link exogenous amines by adding isotopically labeled variants of these compounds to the culture medium. Specifically, we supplemented broth containing *B. fragilis* P207 and deoxycholic acid (0.01% wt/vol) with 1 mM of either isotopically labeled 4-aminobutyric acid (^13^C_4_, 97%–99%), L-alanine (^13^C_3_, 99%), glycine (^13^C_2_, 99%; ^15^N, 98%+), L-phenylalanine (D_8_, 98%), or tyramine:HCl (1,1,2,2-D_4_, 98%). Analyses of spent media from each of these conditions revealed the expected intact mass (MS^1^) and fragments (MS^2^) for all five DCA conjugates (<0.48-ppm error for all MS^1^ ions and <6-ppm error for all MS^2^ ions representing predicted structures), as well as the expected mass shifts when media was supplemented with the isotopically labeled precursors (Fig. 2; Fig. S1 through S5). Given that bile acid conjugation to GABA and tyramine has not been previously reported, we further validated the identity of these compounds by comparing their liquid chromatography retention times and mass spectrometry (MS) data to synthetic standards. These experiments further confirmed the identity of these bile acid amides produced by *B. fragilis* P207 as DCA-GABA and DCA-tyramine (Fig. 3; Fig. S6 and S7). Mass spectrometry evidence for production of these conjugates is fully presented in the Supplemental Results.

**Fig 4:**
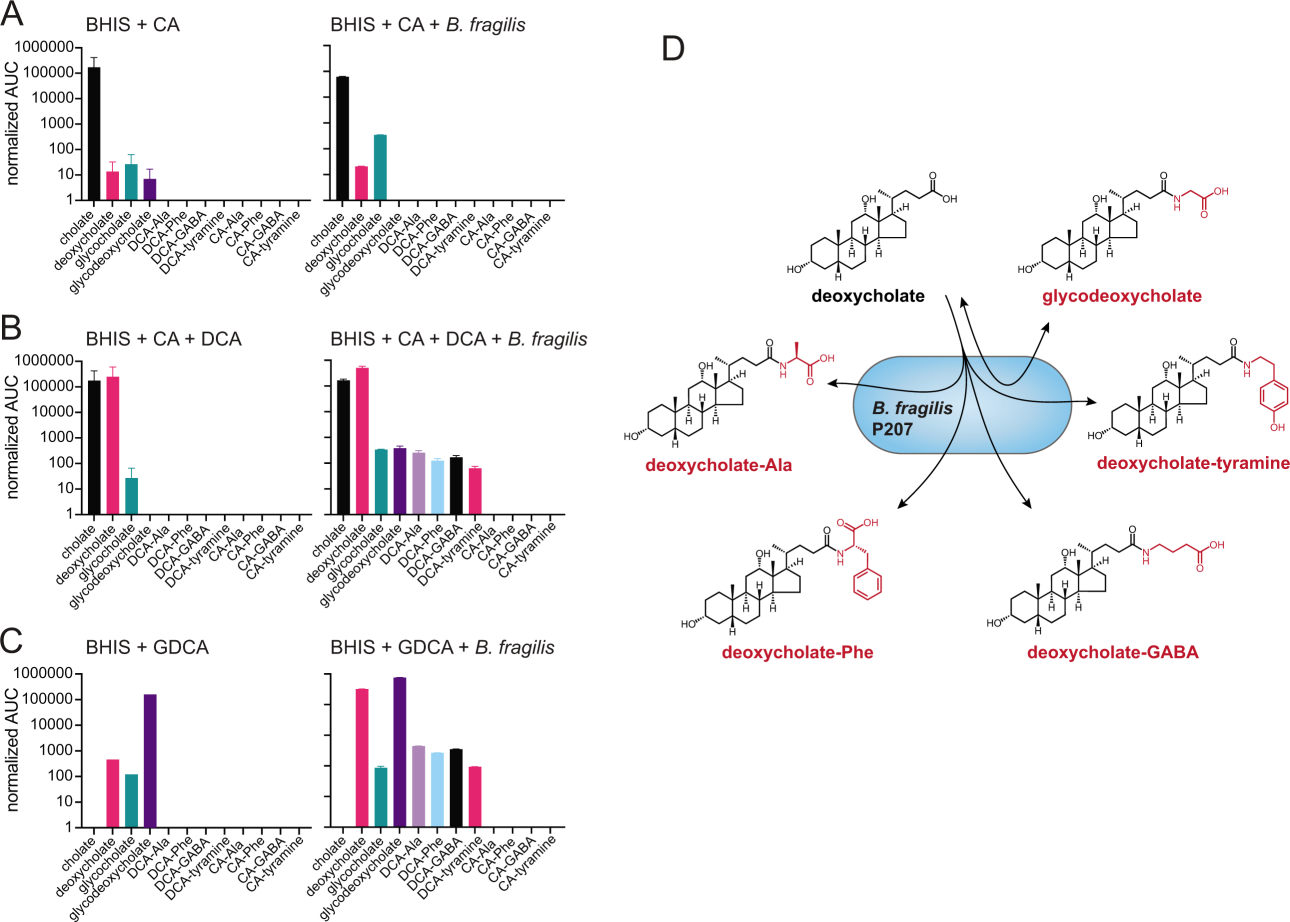
Liquid chromatography-tandem mass spectrometry (LC-MS/MS) measurements of amino acid/amine conjugation to the bile acids cholic acid (CA) and deoxycholic acid (DCA), and evidence for deconjugation/re-conjugation of glycodeoxycholic acid (GDCA) by *B. fragilis* P207. Bar graphs represent the mean area under the curve (AUC) of LC-MS/MS chromatographic peaks corresponding to unconjugated or conjugated bile acids. (**A**) AUC of bile acid conjugate peaks (labeled below each bar) when *B. fragilis* P207 was cultivated in BHIS broth the presence of 0.01% (wt/vol) CA. (**B**) AUC of bile acid conjugate peaks when *B. fragilis* P207 was cultivated in BHIS broth the presence of CA and DCA [0.01% (wt/vol) total]. (**C**) AUC of bile acid and bile acid conjugate peaks from *B. fragilis* P207 culture extract cultivated in BHIS broth the presence of 0.01% (wt/vol) GDCA. Cholate and deoxycholate are known contaminants of the GDCA reagent. Data represent the mean ± SD of two independent cultures. The absence of a bar indicates that a peak corresponding to that chemical species was not detected. (**D**) Cartoon representing the bile acid conjugate production profile of the human gut isolate, *B. fragilis* P207.

To assess the chemical specificity of bile acid conjugation by *B. fragilis* P207, we further tested for production of cholic acid (CA) amides. Mass spectrometry analyses revealed trace levels of DCA in the BHIS growth media and traces of DCA and glycocholic acid (GCA) in the starting CA reagent; GCA levels were enhanced by incubation of CA (0.01% wt/vol) with *B. fragilis* P207 (Fig. 4A). Simultaneous incubation of CA and DCA (0.01% wt/vol total) with strain P207 showed similar production of the same five DCA conjugates described above. Thus, the addition of CA did not apparently impact P207-dependent production of the five DCA conjugates over this timescale (Fig. 4B; Table S2). We conclude that strain P207 prefers DCA as a conjugation substrate over CA in BHIS medium.

Lucas and colleagues have demonstrated that modification of CA into the secondary bile acid, 7-oxo-DCA, is robust and widespread across several species of *Bacteroides*, while production of DCA from CA was limited to *Bacteroides vulgatus* (9). We did not observe increased DCA levels when *B. fragilis* P207 was incubated with CA, nor did we observe production of any DCA conjugates from CA indicating that *B. fragilis* P207 does not catalyze production of DCA from CA under these conditions. The relative abundances of bile acid amide conjugates produced across replicate experiments are presented in Fig. S8.

### P207 catalyzed deconjugation and re-conjugation of amines to DCA

Given that some bacteria can deconjugate glycine and taurine from secreted bile acids, we tested whether *B. fragilis* P207 is able to produce the DCA conjugates described above using glycodeoxycholate (i.e., GDCA) as an initial substrate. *B. fragilis* P207 was incubated in BHIS broth with and without GDCA (0.01% wt/vol), and culture samples were analyzed by UHPLC-MS^2^. We observed trace contaminants DCA and GCA in the growth medium and bile acid reagent, but incubation of GDCA with strain P207 resulted in decreased GDCA and increased DCA levels, providing evidence that *B. fragilis* P207 has GDCA deconjugation activity (Fig. 4C). We further observed production of the DCA conjugates described above (GABA, tyramine, alanine, and phenylalanine) from GDCA when *B. fragilis* was present in the medium (Fig. 4C). We conclude that *B. fragilis* P207 can produce all the DCA conjugates described in the section above when either DCA or GDCA is present as an initial substrate.

### GABA production by *B. fragilis* P207 is induced by deoxycholate

**Fig 5:**
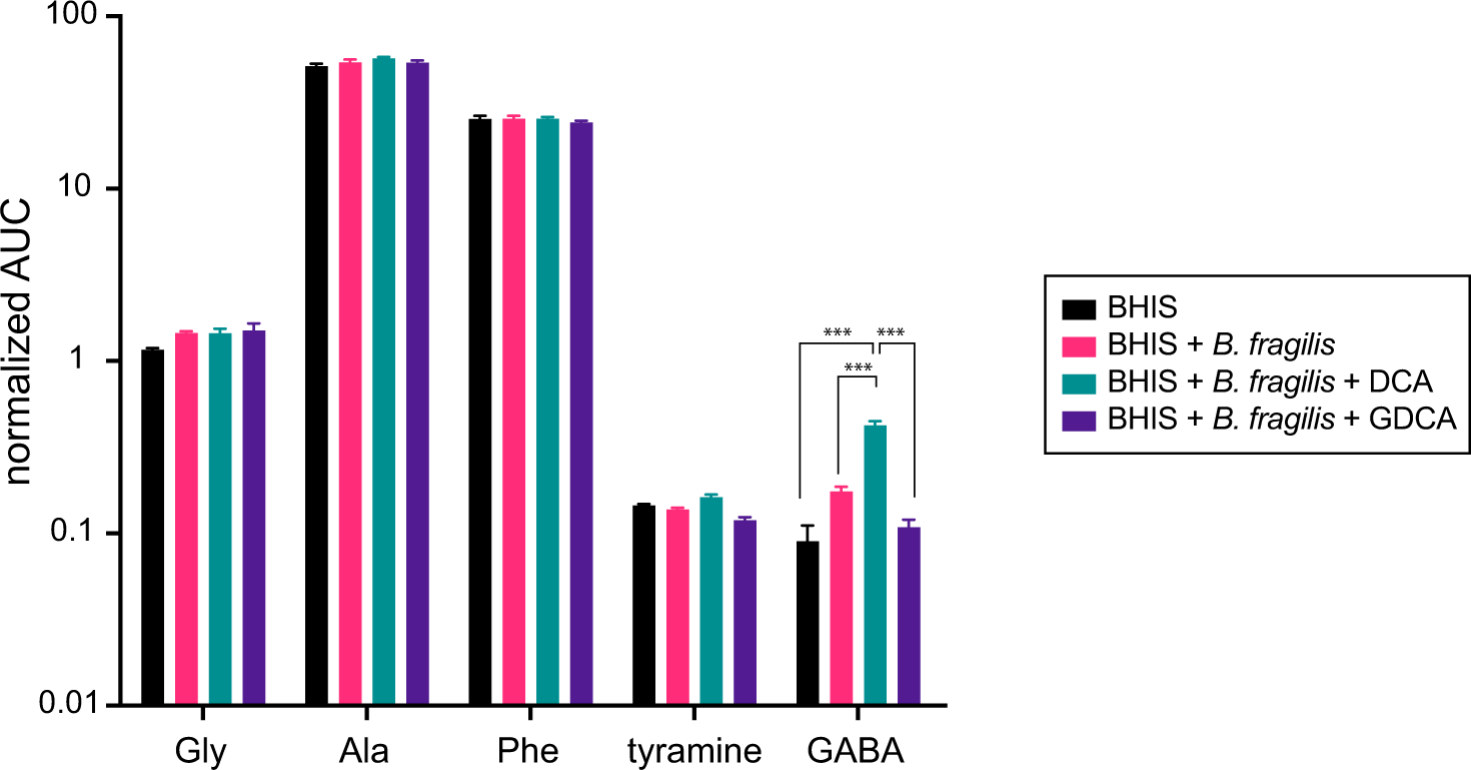
GC-MS-based detection of tyramine, glycine, alanine, phenylalanine, and GABA in *B. fragilis* P207 cultures. Cultures were grown in BHIS medium, both in the absence and presence of bile acids, DCA, and GDCA (0.01% wt/vol). The displayed values, derived from the area under the curve (AUC) of detected peaks, represent the relative concentrations of these amine compounds across the different cultures. Each bar represents the mean of three independent experiments, with error bars indicating the standard deviations. Differences between conditions were assessed for statistical significance using analysis of variance with Bonferroni correction. ***A threshold of *P* < 0.001 was considered to indicate statistical significance.

The observation of bile acid conjugation to a distinct set of amines *in vitro* raised the question about the levels of these amines in the culture medium. To measure the relative levels of specific amine-containing compounds in *B. fragilis* P207 cultures before and after exposure to DCA, we used gas chromatography-mass spectrometry (GC-MS) to analyze the same culture samples in which the DCA conjugates were identified. Among the five amines conjugated to DCA, alanine and phenylalanine were most abundant in the medium, followed by glycine at levels 15–40 times lower. Tyramine and GABA were present at levels approximately 10 times lower than glycine (Fig. 5). Relative amine abundances did not, therefore, directly correlate with the levels of their respective DCA conjugates produced *in vitro* (Fig. S8).

Notably, when DCA was added to the culture medium, a significant increase in GABA was observed; glycodeoxycholic acid (GDCA) addition did not have this effect on microbial GABA production. The relative concentrations of other conjugated amines (i.e., alanine, phenylalanine, and tyramine) was unchanged across all treatment conditions (Fig. 5; Fig. S9). *Bacteroides* species in the human gut are known to produce GABA from either glutamate or glutamine, particularly under pH stress conditions (26). Given the increase in GABA following DCA treatment, we expanded our GC-MS analysis to include glutamine, glutamate, as well as other naturally occurring amino acids. DCA treatment did not impact glutamine levels, but it did lead to a modest reduction in glutamate concentrations (q < 0.01; Fig. S9), which is consistent with a model whereby DCA stimulates *B. fragilis* P207 to convert glutamate into GABA. However, DCA was recently reported to strongly induce expression of glutamate dehydrogenase (gene locus *PTOS_003163*) in *B. fragilis* P207 (27), suggesting that the metabolite flux involving glutamate is likely modulated in multiple ways by DCA. Cultivation of *B. fragilis* P207 did not greatly affect the levels of amino acids in BHIS overall, though glycine, GABA, glutamine, histidine, methionine, proline and tyrosine increased significantly (q < 0.01) after *B. fragilis* was cultivated in the medium. Asparagine and aspartate showed the largest changes in the presence of *B. fragilis*: the concentration of these two amino acids decreased by approximate factors of 50 and 10, respectively (q < 0.001), suggesting they serve as primary nutritional substrates for *B. fragilis* P207 in BHIS medium (Fig. S9).

### Defining the bile acid conjugate profiles of a pouchitis patient and healthy donors

**Fig 6:**
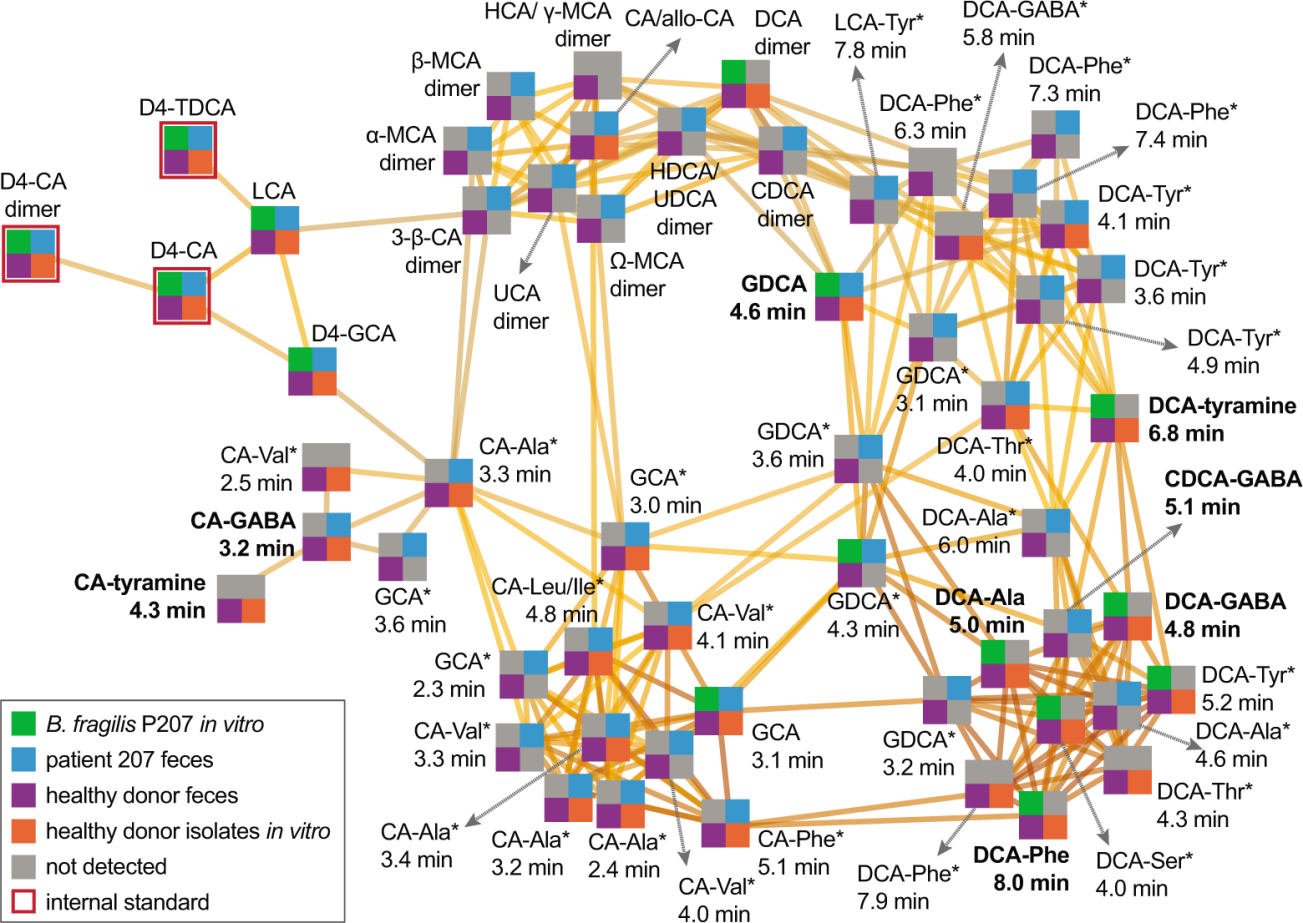
Molecular network illustrating the diversity and occurrence of bile acid-amine conjugates related to the validated DCA-Phe, DCA-Ala, DCA-Gly, DCA-tyramine, DCA-GABA, CDCA-GABA, CA-GABA, and CA-tyramine products. Each node represents a high-resolution *m*/*z* value at a specific retention time. Node quadrant color indicates the sample type that the metabolite was detected in—gray color indicates that the metabolite was not detected in the sample type specified by that quadrant. Edges connect nodes that are related above a score threshold of 25 in Compound Discoverer software suite (Thermo Scientific), with darker edges signifying greater relatedness. Bolded node labels indicate metabolites validated by isotope labeling (GDCA, DCA-Ala, and DCA-Phe) and/or comparison to true synthetic standards (DCA-GABA, DCA-tyramine, CDCA-GABA, CA-GABA, and CA-tyramine). Labels with an asterisk (*) indicate that the node represents a putative bile acid-amine isomer/epimer of the listed metabolite. These isomer assignments are based on comparison of *m*/*z* of the intact ion and MS^2^ fragmentation spectra and fragment *m*/*z* values to the spectra of the validated bile acid conjugates. All other metabolite nodes were confirmed by comparison to authenticated standards or labeled internal standards. D4 indicates deuterated standards. CA, cholic acid; CDCA, chenodeoxycholic acid; DCA, deoxycholic acid; GCA, glycocholic acid; HCA, hyocholic acid; HDCA, hyodeoxycholic acid; LCA, lithocholic acid; MCA, muricholic acid; TDCA, taurodeoxycholic acid; UCA, ursocholic acid; UDCA, ursodeoxycholic acid.

**Fig 7:**
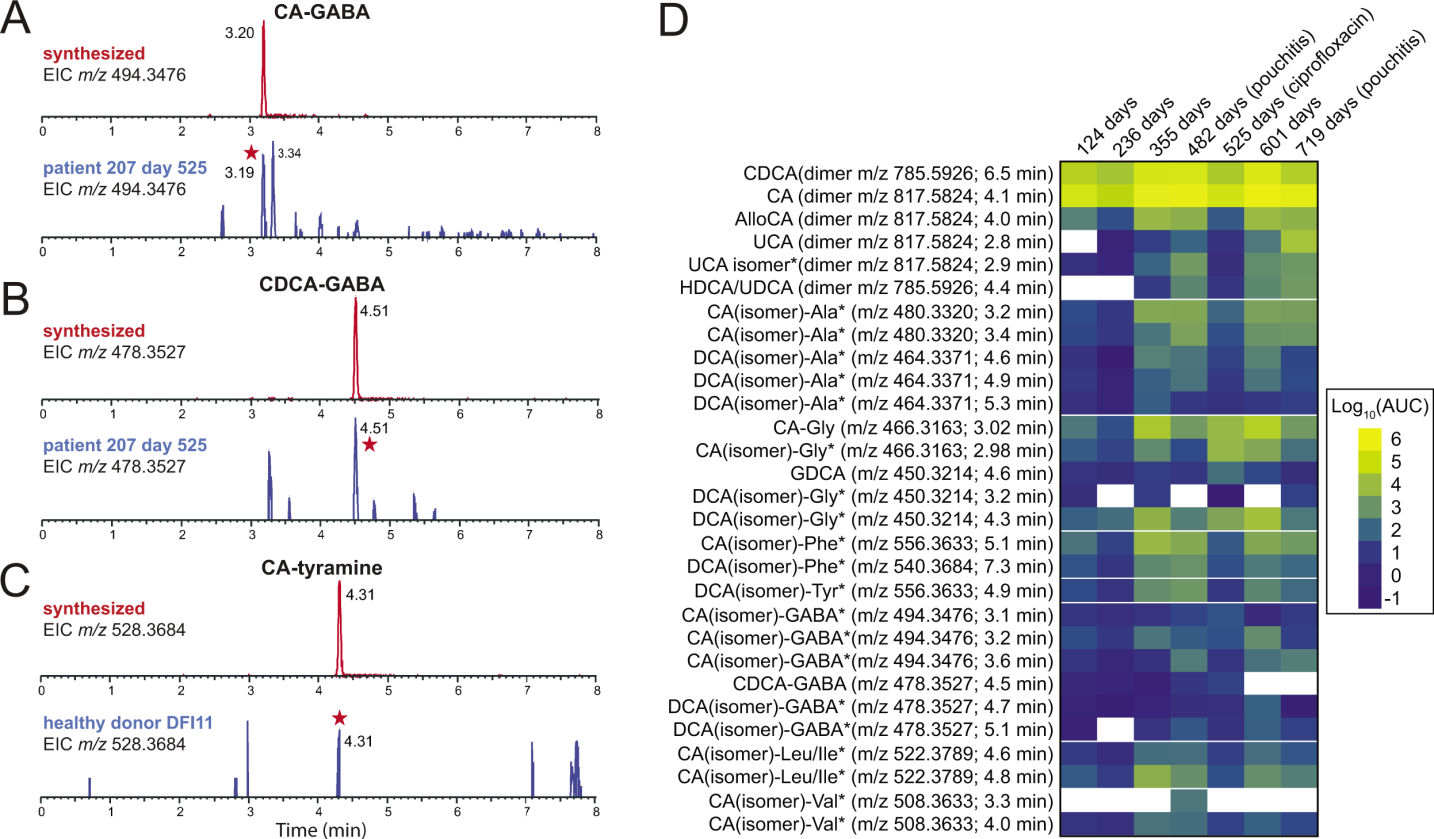
Extracted ion chromatograms (EICs) of synthetic standards of cholic acid (CA) or chenodeoxycholic acid (CDCA) conjugated to GABA or tyramine compared to stool sample extracts. (**A**) EIC of chemically synthesized CA-GABA (*m*/*z* 494.3476) (top, retention time = 3.20 min) and the corresponding EIC from patient 207 stool at 525 days post functionalization of the ileal pouch (bottom). Peak matching synthetic CA-GABA is marked with a red star. (**B**) EIC of chemically synthesized CDCA-GABA (*m*/*z* 478.3527) (top, retention time = 4.51 min) and the corresponding EIC from patient 207 at 525 days post functionalization of the ileal pouch (bottom). Peak matching CDCA-GABA is marked with a red star. (**C**) EIC of chemically synthesized CA-tyramine (*m*/*z* 528.3864) (top, retention time = 4.31 min) and the corresponding EIC from healthy patient donor (DFI11) stool (bottom). Peak matching synthetic CA-tyramine is marked with a red star. (**D**) Heatmap illustrating the levels of unconjugated and conjugated bile acids in pouchitis patient 207 (measured by area under the curve) from days 124 to 719 after J-pouch functionalization. DCA (3α,12α-dihydroxy-5β-cholan-24-oic acid) was absent at all time points in this patient, and CDCA was abundant, so we expect the labeled DCA (isomer) amide conjugates have a CDCA core, though the isomeric/epimeric form is not defined in most cases. Likewise, we expect that many of the abundant CA (isomer) conjugates have a cholic acid core but the particular isomer/epimer of these products has not been defined.

To test if the bile acid conjugates produced by *B. fragilis* P207 *in vitro* were present in the gut of the human patient from whom the strain was isolated, we prepared extracts of stool samples collected from patient 207 at time points before and after onset of pouchitis and during antimicrobial treatment (23). Metabolomic data from this patient were compared to UHPLC-MS^2^ data from 21 healthy donor stool samples. Patient 207 and healthy donor stool contained a complex mixture of bile acids (Fig. 6; Table S2). Unlike healthy donors, which contained expected high levels of DCA, patient 207 lacked DCA across all time points (Fig. S10; Table S2). This result is congruent with a reported deficiency in secondary bile acids in UC pouches (22). The primary bile acids chenodeoxycholate (CDCA) and CA were abundant across all time points in patient 207, at levels that were ≈60 times higher on average than observed in the 21 healthy donor samples (*P* < 0.001) (Fig. S11; Table S2). Amine conjugates to CA and CDCA, including putative GABA conjugates, were identified in patient 207 stool (Fig. 6 and 7; Fig. S12 and S13; Table S2). We detected several amines conjugated to a bile acid core with an *m*/*z* corresponding to DCA or its isomers (Fig. 6; Fig. S8). Considering the lack of DCA and abundance of CDCA in patient 207, we predicted that these species were CDCA derivatives. Indeed, a product with an elution profile and MS^2^ fragmentation pattern matching a CDCA-GABA synthetic standard was identified in patient 207 stool (Fig. 7; Fig. S12). We further identified products matching CA-GABA and CA-tyramine synthetic standards in patient 207 stool and in healthy human donor stool, respectively (Fig. 7; Fig. S13 and S14). The results provide strong evidence that bile acids conjugated to GABA and tyramine are present in the human gut.

*B. fragilis* strain P207 dominated the pouch ecosystem of patient 207 from 182 to 434 days after surgical functionalization of the ileal J-pouch (i.e., IPAA) (23). Normalized levels of bile acids, including the primary bile acids CA and CDCA, were lowest at 124 and 236 days after IPAA. There was a marked increase in unconjugated CA and CDCA and bile acid conjugates at 355 days post-functionalization, and levels trended upward by 482 days when the patient was diagnosed with pouchitis and initiated a course of antibiotic therapy (ciprofloxacin) (Fig. 7D; Fig. S11). The contribution of *B. fragilis* strain P207 to the production of particular bile acid conjugates at these time points is not known, but the *in vitro* conjugation data presented above indicate that CA conjugates (other than glycocholate) are not produced by *B. fragilis* P207 (Fig. 4). At a follow-up visit 525 days post-IPAA, ciprofloxacin treatment had resulted in a marked reduction of the pouch microbiome census, and pouch inflammation was resolved (23). Bile acid analysis showed that levels of conjugated and unconjugated bile acids were sharply reduced at this time point except for select low abundance conjugates of CA and DCA isomers to glycine and GABA, which increased (Fig. 7D; Fig. S11 and S15; Table S2). Though inflammation was resolved by ciprofloxacin treatment, inflammation returned by 719 days (23). In this period, the dominant species of the pouch shifted from *B. fragilis* P207 to *Bacteroides ovatus* (23). This was correlated with a change in the bile acid conjugate profile, with distinct CA and DCA isomer conjugates to GABA and glycine increasing (Table S2; Fig. S11 and S15)

### Conjugation of GABA and tyramine to CA by human gut isolates

**Fig 8:**
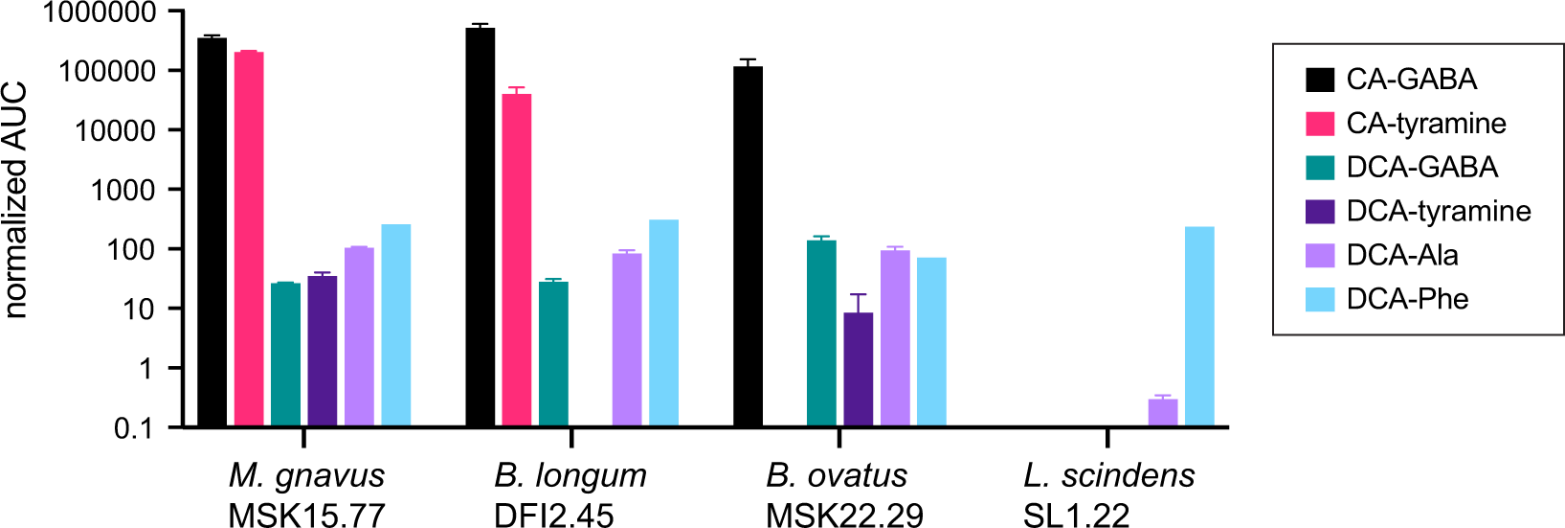
UHPLC-MS^2^ measurements of amino acid/amine conjugation to the bile acids cholic acid (CA) and deoxycholic acid (DCA) by *M. gnavus*, *B. longum*, *B. ovatus*, and *L. scindens* strains isolated from healthy human patients. The bar graphs in this figure represent the mean area under the curve (AUC) of LC-MS/MS peaks corresponding to unconjugated or conjugated CA and DCA (*n* = 2, errors bars are standard deviations). Normalized AUC of bile acid conjugate peaks (colored according to key) when strains were cultivated in BHIS broth in the presence of 0.01% (wt/vol) DCA or CA. The absence of a bar indicates that a peak corresponding to that chemical species was not detected.

Metabolomic analysis of stool samples from pouchitis patient 207 and healthy human donors revealed a complex mixture of known and previously unreported bile acid conjugates, including CA conjugates to GABA and tyramine (Fig. 6 and 7). To discover bacteria that can produce these novel CA conjugates, we inspected the genomes of a collection of human gut isolates for genes that encode predicted N-terminal nucleophilic cysteine hydrolase (Ntn) enzymes, which includes known choloylglycine hydrolases [Conserved Domain Database accession cd01902 (28)]. Recent studies report that this class of enzymes can function to both deconjugate and conjugate bile acids (29, 30). *B. fragilis* P207 encodes an Ntn hydrolase (WP_005817456.1), and we predicted that other strains encoding these enzymes may conjugate GABA or tyramine to CA *in vitro*. We selected a phylogenetically diverse group of human patient isolates that were also predicted to encode an Ntn hydrolase, including *Mediterraneibacter gnavus* MSK15.77 (WP_004614568.1), *Bifidobacterium longum* DFI.2.45 (WP_007052221.1), and *Bacteroides ovatus* MSK22.29 (which encodes three Ntn paralogs: WP_217723859.1, WP_004308262.1, and WP_004323538.1) (Fig. S15). We further identified a strain in our collection that does not encode a predicted Ntn bile salt hydrolase, *Lachnoclostridium scindens* SL.1.22. All three Ntn-encoding gut isolates produced putative GABA conjugates to CA, and *M. gnavus* and *B. longum* cultures contained a product that had an MS^2^ fragmentation pattern consistent with CA-tyramine (Fig. 8; Fig. S16). *L. scindens* cultures did not contain CA conjugates.

We further investigated if these strains conjugated amines to DCA and detected conjugates of alanine, phenylalanine, and GABA to DCA in the culture extracts of *M. gnavus*, *B. longum*, and *B. ovatus*. Additionally, DCA-tyramine conjugates were identified in *M. gnavus* and *B. ovatus* cultures (Fig. 8). Although the *L. scindens* SL.1.22 genome does not encode a predicted Ntn family/bile salt hydrolase (BSH) enzyme, we observed low levels of DCA-Phe and DCA-Ala conjugates in its culture extract when this strain was incubated with 0.01% (wt/vol) DCA. The specific genes responsible for DCA conjugation in our *L. scindens* cultures remain unidentified.

## DISCUSSION

### On the mechanism of microbial bile acid conjugation

*B. fragilis* P207 produces a suite of DCA amides *in vitro*, in which the bile acid carboxylate group is conjugated to the amine group of select amino acids and neuroactive amines. Biosynthesis of bile acid amides by gut microbes is a recent discovery (9, 10), and there is now genetic and biochemical evidence that bacterial BSH enzymes can catalyze aminoacyl transfer to bile salts (29, 30). Thus, BSH proteins have significant N-acyl transferase activity in addition to their long-established function as hydrolases (31). While microbial conjugation of bile acids to the α amino group of amino acids has been described, our data provide evidence that an activity encoded by *B. fragilis* P207 enables conjugation of the primary amine groups of GABA and tyramine to DCA. DCA conjugate biosynthesis was evident when culture broth was supplemented with either unconjugated (DCA) or conjugated (GDCA) bile acids, which supports a model in which P207 first deconjugates GDCA and then generates DCA amides from the deconjugated product.

The *B. fragilis* P207 genome (GenBank accession CP114371) encodes a single predicted BSH (gene locus *PTOS_003312*) that shares 80% identity with BT2086, a protein that has demonstrated BSH activity in *Bacteroides thetaiotaomicron* (32). PTOS_003312 is 99% identical to the BSH of *B. fragilis* strain 638R (locus *BF638R_3310*), which promotes deconjugation of primary bile acids in the gut of germ-free mice (33). In light of recent *in vitro* biochemical data showing that purified *Clostridium perfrigens* (29) and *Bifidobacterium longum* (30) BSH enzymes produce bile acid amides from both unconjugated (CA) and conjugated [taurocholic acid (TCA)] bile acids, it seems most likely that PTOS_003312 of *B. fragilis* P207 catalyzes the production of the five bile acid conjugates reported here, using either GDCA or DCA as substrates, though we cannot rule out the possibility that these products arise from the activity of multiple enzymes. P207 demonstrates a clear preference for bile acid amide production from the secondary bile acid, DCA, over primary bile acid CA, which differ only by a single hydroxyl group at C-7. Based on this result, we infer that the C-7 position of the sterol core is important for bile acid substrate interaction with the transforming enzyme(s) of strain P207.

The detection of DCA conjugation to the primary amine groups of GABA and tyramine raises questions about the mechanism of bile acid conjugation, given the relative difference in nucleophilicity and steric accessibility of the primary amine groups of tyramine and GABA (pKa ≈ 10.5–11.0 in aqueous solvent) and the typical α amino group (pKa ≈ 9–9.5) of amino acids. Considering the diversity in primary structure of BSH enzymes and the selectivity of BSH enzymes for particular steroidal cores across *Bacteroides* spp. (32), it is likely that structural differences in the active site or other regions of the conjugating enzyme (34) will determine whether primary amine groups, such as those on GABA and tyramine, form amide bonds with bile acid(s). Evidence for microbially catalyzed production of GABA and tyramine BA amides presented herein is congruent with a recent report that a diverse array of amines in the gut are conjugated to bile acids (35).

The benefit of bile acid conjugation to *B. fragilis* and other microbes studied here, if any, is not known. *B. fragilis* P207 can deconjugate GDCA and produce a variety of conjugated compounds from the deconjugated bile acid substrate (DCA) (Fig. 4). Unconjugated primary and secondary bile acids are often more toxic *in vitro* than the amino acid-conjugated forms (13, 36, 37). Indeed, we have shown that *B. fragilis* P207 is more sensitive to CA and DCA than glycocholate and glycodeoxycholate (Fig. S17). We further observed that the secondary bile acid, DCA, is more toxic to *B. fragilis* P207 than CA. The ability to conjugate amino acids effectively detoxifies DCA *in vitro* and may therefore influence the impact of bile salts on *B. fragilis* physiology in certain settings.

### Broader connections of bile acid conjugation to mammalian physiology

This study defines the temporal bile acid profile of the J-pouch of a pouchitis patient before, during, and after inflammation, including a period of antibiotic therapy, and draws comparisons of this profile to stools of healthy individuals. Healthy human donors presented expected high levels of DCA, while the pouchitis patient was severely DCA deficient. This aligns with previous observations of reduced secondary bile acids in UC pouches (22). As expected for a patient with diminished secondary bile acid production, the primary bile acids, CDCA and CA, were significantly elevated, and unique amine conjugates to CA and CDCA, particularly novel GABA conjugates, were identified in this patient. Our *in vitro* analyses of *B. fragilis* P207 and of other human gut microbiome isolates have demonstrated that bile acid conjugates to GABA and tyramine observed in stool can be produced by multiple bacterial genera (Fig. 8). Changes in the levels of bile acids leading up to pouchitis, coupled with the marked influence of ciprofloxacin treatment on the bile acid profile (Fig. S9 and S12), underscore the complex interplay between gut microbiota, host factors, and therapeutic interventions on the bile acid profile of humans.

The neuroactive amines, GABA and tyramine, are potent regulators of mammalian physiology and both molecules are produced by microbes that comprise the gut microbiome, including Bacteroidetes (26). GABA has a well-recognized role in the regulation of gut physiology (38). GABA-producing *Bacteroides* are reported to increase steady-state levels of GABA in the intestines of mono-associated germ-free mice (39), and we have shown that bile acid exposure enhances *B. fragilis* GABA production *in vitro* (Fig. 5) and provided evidence that exogenous GABA can be conjugated by *B. fragilis* P207 (Fig. 2). The conjugation and subsequent uptake of bile acid-GABA amides into enterohepatic circulation may impact GABAergic signaling in the gut and possibly at other distal tissue sites. Tyramine is a product of tyrosine catabolism but is also present at high levels in a variety of foods. Steady-state tyramine levels in human tissue are typically low due to the activity of monoamine oxidase A but can be modulated in patients treated with monoamine oxidase inhibitors (40). Trace amine-associated receptors (TAARs), with which tyramine can interact, have been reported in enterochromaffin cells of the human gut (41), though the impact of dietary and microbiome-derived tyramine of TAAR signaling in the gut is not known. Tyramine-bile acid amides in human patients likely vary as a function of diet and other factors and vary geographically along the digestive tract, depending on the amount of microbial tyramine production and the spectrum of bile acid conjugating activity conferred by the host microbiome at particular foci. Tyramine feeding data presented in Fig. 2 suggest that dietary tyramine could impact BA-tyramine levels in the gut.

The impact of specific conjugated bile acids reported here on signaling through bile acid receptors is an interesting future area of investigation. BSH activity of non-enterotoxigenic *Bacteroides* spp. was recently reported to potentiate obesity-related colorectal cancer progression in a mouse model (42); this effect was attributed in part to enhanced TGR5 signaling as a result of increased levels of deconjugated DCA and lithocholic acid in the colon. Certainly, gut microbe BSH activity can lower the level of deconjugated bile acids in the gut. However, it is important to consider that BSH activity results in a spectrum of unconjugated and conjugated bile acids (29, 30), including compounds reported here. The effect of these compounds on signaling from bile acid receptors may shape host physiology, disease, and health.

## MATERIALS AND METHODS

### Cultivation of bacteria in the presence of bile acids

*B. fragilis* P207 (NCBI accession NZ_CP114371) was cultivated in BHIS containing either 0.01% (wt/vol) DCA, CA, or GDCA that was inoculated from a saturated starter culture (≈1.0 OD_600_), back-diluted 1:10. Cultures were grown for 24 h; 1 mL of culture was removed and flash frozen for subsequent metabolomic analysis. *Mediterraneibacter gnavus* MSK15.77 (NCBI accession NZ_JAAIRR010000000), *Bacteroides ovatus* MSK22.29 (NCBI accession NZ_JAHOCX010000000), *Bifidobacterium longum* DFI.2.45 (NCBI accession NZ_JAJCNS010000000), and *Lachnoclostridium scindens* SL.1.22 (NCBI accession GCA_020555615.1)were also cultivated in BHIS. Briefly, starter cultures were inoculated from frozen glycerol stocks and grown anaerobically at 37°C overnight to ≈1.0 OD_600_. These cultures were diluted to ≈0.005 OD_600_ in 1 mL of plain BHIS or BHIS containing 0.01% (wt/vol) DCA or CA in a 96-well deep well plate. The plate was incubated at 37°C anaerobically for 20 h and then frozen at −80°C for subsequent metabolomic analysis.

### Untargeted metabolomic approach to detect bile acid conjugates

Bacteria culture supernatants were lyophilized followed by 2× concentration in 100% methanol (containing internal standards). Samples were then centrifuged at −10°C, 20,000 × *g* for 15 min to generate supernatants for subsequent metabolomic analysis. All ultra-high-performance-liquid chromatography-mass spectrometry (UHPLC-MS) analyses were performed using a Thermo Scientific Vanquish Flex UHPLC coupled with an IQ-X mass spectrometer (Thermo Fisher Scientific). Reversed-phase chromatography was performed on a Waters CORTECS T3 C18 RP-UHPLC column [100 × 2.1 mm inner diameter, 1.6-µm particle size, 120-Å pore size (1 Å = 0.1 nm)]. Mobile phase A was 5% acetonitrile in water with 0.1% formic acid, and mobile phase B was acetonitrile with 0.1% formic acid. For the initial analysis of *B. fragilis* P207 cultures (as displayed in Fig. 1), the flow rate was 550 µL/min. The chromatographic method used was an isocratic 20% mobile phase B for 0.2 min, followed by a gradient of 20%–97% mobile phase B for 8.7 min with a wash of 97% mobile phase B for 1.0 min. Analysis of *B. fragilis* P207 cultures for normalized relative quantification (as displayed in Fig. S8) was performed at a flow rate of 480 µL/min. The chromatographic method used was an isocratic 100% mobile phase A for 0.2 min, followed by a gradient of 0 to 97% mobile phase B for 10.2 min with a wash of 97% mobile phase B for 1.0 min. Optimized chromatography was then developed for all other UHPLC-MS/MS experiments to better separate the numerous isomers of the various bile acid amine conjugates. The chromatographic method used a flow rate of 300 µL/ min with a gradient of 20% to 40% mobile phase B for 0.2 min, followed by a gradient of 40%–70% mobile phase B for 11.8 min, a gradient of 70%–97% mobile phase B for 0.1 min, and a wash of 97% mobile phase B for 1.1 min.

All samples were analyzed using positive ionization. Flow from the UHPLC was ionized with the heated electrospray ionization source set to 3,400 V; ion transfer tube temperature set to 200°C; vaporizer temperature set to 400°C; and sheath, aux, and sweep gases set to arbitrary values of 40, 5, and 1, respectively. Data for MS^1^ were acquired using a maximum inject time of 50 ms, a normalized AGC target of 25%, a 300–1,700 *m*/*z* quadrupole scan range, and a resolution of 60,000. All tandem MS^2^ mass spectral data were acquired using a 1.5 *m*/*z* quadrupole isolation window, a maximum inject time of 22 ms, a normalized AGC target of 20%, and a resolution of 15,000. Each of the metabolite ions of interest was added to an inclusion list for MS^2^ fragmentation by a normalized higher-energy collisional dissociation energy of 30%. Data analysis was performed using FreeStyle software (version 1.8 SP2, Thermo Scientific), MZmine (version 2.53) (43), Global Natural Products Social Molecular Networking (GNPS) platform tools Feature-Based Molecular Networking (FBMN) release 28.2, and Mass Search Tool release 29 (44 – 46), and GraphPad Prism (version 9.4.1 for Windows; GraphPad Software, San Diego, California, USA; www.graphpad.com). Areas under the curve (AUCs) were normalized to the average of D_4_-taurocholate and D_4_-taurodeoxycholate areas under the curve and multiplied by 1,000:

Normalized AUC = {peak_AUC_ / [(D_4_ − TC_AUC_ + D_4_ − TDC_AUC_) / 2]} × 1,000

### Data processing and molecular networking

Data-dependent mass spectrometry data files were processed using MZmine 2.53 and the Feature-Based Molecular Networking function in the GNPS environment to identify and network spectral features while also matching generated data to publicly available library spectra. MZmine was used to detect MS^1^ and MS^2^ peaks, build deconvoluted extracted ion chromatograms, group isotopes, and match the resulting features across samples, accounting for retention time drift across injections. Features within each sample were then normalized by dividing peak area by the average peak area of the internal standards in that sample. Features that are found in solvent and method blank controls were then eliminated. The aligned feature lists were then exported for analysis using the FBMN tool within the GNPS platform.

FBMN was performed with precursor ion mass tolerance and fragment ion mass tolerance both set to 0.02 Da, minimum matched fragment ions set to six, networking cosine score set to >0.7, library cosine score set to >0.7, and minimum library shared peaks set to six. A visualization of the network was constructed in Cytoscape by drawing edges between scan nodes with a cosine similarity of >0.7. The network was manually analyzed to identify ions that occurred only in samples containing BHIS, a bacterial strain, and DCA or CA, with a particular focus on those that occurred within the same molecular families as our bile acid internal standards and other nodes that matched to library entries of known bile acids. Nodes in the molecular network were also checked against a list of predicted *m*/*z* values for hypothesized amine-containing conjugates presented in Table S1. Data files were also manually analyzed for MS^2^ scans containing characteristic DCA core fragments at *m*/*z* 215.1794 and *m*/*z* 339.2682 (observable in Fig. S1 through S5) to identify metabolite ions that may not have been clustered in the molecular network due to falling below networking thresholds.

### Isotopically labeled amine feeding experiments

To test for conjugation of isotopically labeled amines to DCA by *B. fragilis* P207, 1-mL cultures containing 1 mM (final concentration) of either 4-aminobutyric acid (^13^C4, 97%–99%), L-alanine (^13^C3, 99%), glycine (2-^13^C, 99%; ^15^N, 98%+), L-phenylalanine (D_8_, 98%), or tyramine:HCl (1,1,2,2-D_4_, 98%) (Cambridge Isotope Laboratories, Inc.) were added to BHIS containing 0.1% (wt/vol) DCA (Thermo Fisher Scientific). Control samples contained either no isotopically labeled amines, no DCA, or BHIS media without any additional supplement. Culture supernatants were prepared for metabolomic analysis as described above.

### Synthesis of bile acid conjugate standards

Synthesis of bile acid-amine conjugates followed methods previously described (10). Cholic acid, deoxycholic acid, and chenodeoxycholic acid were dissolved to 0.125 mM in 5-mL anhydrous THF (tetrahydrofuran), each in two separate 20-mL scintillation vials containing stir bars, and kept on an ice bath for a total of six vials. Next, ethyl chloroformate (0.15 mmol, 7 µL) and triethylamine (0.15 mmol, 10.5 µL) were added to each reaction vessel and stirred for 1.5 h in the ice bath. Basic aqueous solutions of GABA (1.54 eq, 0.193 mM) and tyramine (1.54 eq, 0.193 mM) were prepared using NaOH (1.5 eq, 0.188 mM), with 5 mL of the GABA solution added to the first of each pair of bile acid solutions and 5 mL of the tyramine solution added to the second of each pair of bile acid solutions. The reaction mixtures were stirred for 2 h, centrifuged at −10°C, 20,000 × *g* for 15 min to remove any remaining insoluble reaction components, and injected on the UHPLC-MS/MS using the optimized bile acid chromatography method.

### Metabolite analysis using GC-electron ionization-MS with methoxyamine and trimethylsilyl derivatization

Amine-containing metabolites were measured using GC-MS with electron impact ionization. Bacterial cultures were extracted using four volumes of 100% methanol. Following brief vortexing and centrifugation at 4°C, 20,000 × *g* for 15 min, we added 100 µL of extract supernatant to prelabeled mass spec autosampler vials (microliter, 09–1200) and dried down completely under nitrogen stream at 30 L/min (top), 1 L/min (bottom) at 30°C (Biotage SPE Dry 96 Dual, 3579M). To dried samples, 50 µL of freshly prepared 20-mg/mL methoxyamine (Sigma, 226904) in pyridine (Sigma, 270970) was added and incubated in a thermomixer C (Eppendorf) for 90 min at 30°C and 1,400 rpm. After samples are cooled to room temperature (RT), 80 µL of derivatizing reagent (BSTFA + 1% TMCS; Sigma, B-023) and 70 µL of ethyl acetate (Sigma, 439169) were added, and samples were incubated in a thermomixer at 70°C for 1 h and 1,400 rpm. Samples were cooled to RT and 400 µL of ethyl acetate was added to dilute samples. Turbid samples were transferred to microcentrifuge tubes and centrifuged at 4°C, 20,000 × *g* for 15 min. Supernatants were then added to mass spec vials for GC-MS analysis. Samples were analyzed using a GC-MS (Agilent 7890A GC system, Agilent 5975 MS detector) operating in electron impact ionization mode, using an HP-5MSUI column (30 m × 0.25 mm, 0.25 µm; Agilent Technologies 19091S-433UI) and 1-µL injection. Oven ramp parameters were 1 min hold at 60°C, 16°C/min up to 300°C with a 7 min hold at 300°C. Inlet temperature was 280°C and transfer line was 300°C. Data analysis was performed using MassHunter Quantitative Analysis software (version B.10, Agilent Technologies) and confirmed by comparison to authentic standards. Normalized peak areas were calculated by dividing raw peak areas of targeted analytes by averaged raw peak areas of ^15^N,d_7_-L-proline and U-^13^C-palmitate internal standards.

### Bile acid analysis of human stool

Patient 207 feces was liquid and extraction was performed by vortexing and diluting 1:2 (200 µL into 400 µL) with 100% methanol containing internal standards, followed by bath sonication for 10 min. Non-diseased volunteer donor feces were solid and were extracted by adding 80% methanol to 100 mg/mL and stored at −80°C for at least 1 h in beadruptor tubes (Fisherbrand, 15–340-154). Donor samples were then homogenized at 4°C on a Bead Mill 24 homogenizer (Fisher, 15–340-163) set at 1.6 m/s with six 30-s cycles, 5 s off per cycle. All samples were then centrifuged at −10°C, 20,000 × *g* for 15 min to generate supernatants for subsequent metabolomic analysis. Areas under the curve for culture supernatants were normalized to the average of D_4_-taurocholate and D_4_-taurodeoxycholate areas under the curve and multiplied by 1,000, while areas under the curve for fecal samples were normalized to the average of D_4_-taurocholate and D_4_-glycocholate areas under the curve and multiplied by 1,000. All peak assignments in stool samples were verified by comparing the MS^2^ fragmentation spectra to MS^2^ fragmentation spectra established from the *in vitro* analyses described above.

### *B. fragilis* P207 bile sensitivity growth assays

*B. fragilis* strain P207 was cultivated in BHIS supplemented with increasing concentrations of DCA, CA, GDCA, or GCA. Concentrations ranged from 0.024 to 6.17 mM of each compound. Strain P207 was grown from a saturated starter culture (~1.0 OD_600_) that was back-diluted 1:100. Cultures were then grown for 24 h, at which point terminal culture density was measured at OD_600_.

## Data Availability

All mass spectrometry data files for this study have been submitted to the MassIVE data repository in raw and open source formats under accession numbers MSV000093027, MSV000093028, MSV000093029, MSV000093030, MSV000093031, MSV000093032, MSV000093035, MSV000093039, and MSV000093695. The Global Natural Products Social Molecular Networking (GNPS) Feature-Based Molecular Networking job used to aid in the identification of the deoxycholic acid amine conjugates from the initial untargeted screen of *Bacteroides fragilis* P207 cultures can be accessed on the GNPS platform.

## SUPPLEMENTAL MATERIAL

Supplemental material jb.00426-23-s0001.pdf Supplemental results, Table S1, and Figures S1 to S17.

Table S2 jb.00426-23-s0002.xlsx BA metabolomic data.

ASM does not own the copyrights to Supplemental Material that may be linked to, or accessed through, an article. The authors have granted ASM a non-exclusive, world-wide license to publish the Supplemental Material files. Please contact the corresponding author directly for reuse.
