# Supplementary material for: Microbially catalyzed conjugation of GABA and tyramine to bile acids
Source: J Bacteriol. 2024 Jan 4;206(1):e00426-23. doi: 10.1128/jb.00426-23 (PMC10810215; doi:10.1128/jb.00426-23)
Supplement: Supplemental material — Supplemental results, Table S1, and Figures S1 to S17. [file jb.00426-23-s0001.pdf]

Supplemental Results

Microbially-catalyzed conjugation of GABA and tyramine to bile acids

Michael W. Mullowney, Aretha Fiebig, Matthew K. Schnizlein, Mary McMillin, Amber R. Rose, Jason Koval, David Rubin, Sushila Dalal, Mitch Sogin, Eugene B. Chang, Ashley M. Sidebottom, Sean Crosson

Mass spectrometry data

Glycine conjugate

Broth from P207 cultures containing DCA and unlabeled glycine or isotopically labeled glycine ($^{13}C_2$, 99%; ^{15}N, 98%+) revealed a DCA-Gly conjugate with an $[M+H]^+$ of *m/z* 450.3220 and a corresponding labeled ion with an $[M+H]^+$ of *m/z* 452.3230, respectively (Figure 2A). Analysis of tandem MS fragmentation indicated condensation of the amine of Gly with DCA at the terminal side-chain carboxylic acid (Figure S1). Key MS^2 fragments from unlabeled P207 culture samples that indicate the position of the glycine on DCA include the doubly dehydroxylated DCA-Gly conjugate ion (*m/z* 414.3007), the *N*-(1-Oxo-4-penten-1-yl)glycine ion (*m/z* 158.0807), and the glycine aminium ion (*m/z* 76.0393). Ions with two Dalton increases corresponding to these were observed in MS^2 data associated with an MS^1 scan with a two Dalton increase in the glycine ($^{13}C_2$, 99%; ^{15}N, 98%+) labeled sample, further confirming the structural assignment of glycine conjugation at the terminal side-chain carboxylic acid of DCA.

Alanine conjugate

Broth from strain P207 cultures containing DCA and unlabeled L-alanine or isotopically labeled L-alanine ($^{13}C_3$, 99%) revealed a DCA-Ala conjugate with an $[M+H]^+$ of *m/z* 464.3373 and a

corresponding labeled ion with an $[M+H]^+$ of *m/z* 467.3475, respectively (Figure 2B). The tandem MS fragmentation profile indicated condensation of the amine of Ala with DCA at the terminal side-chain carboxylic acid (Figure S2). Key MS^2 fragments from unlabeled P207 cultures that support the position of the alanine on DCA include the doubly dehydroxylated DCA-Ala conjugate ion (*m/z* 428.3165), the *N*-(1-Oxo-4-penten-1-yl)alanine ion (*m/z* 172.0963), and the alanine aminium ion (*m/z* 90.0544). Ions with three Dalton increases corresponding to the dehydroxylated DCA-Ala conjugate and the alanine aminium ion were observed in MS^2 data associated with an MS^1 scan with a three Dalton increase in the L-alanine ($^{13}C_3$, 99%) sample. These results further support the structural assignment of alanine conjugation at the terminal side-chain carboxylic acid of DCA, which was recently described using an alternative derivatization method (1).

Phenylalanine conjugate

Comparison of broth from P207 cultures containing DCA and unlabeled L-phenylalanine or isotopically labeled L-phenylalanine (D_8, 98%) revealed a DCA-Phe conjugate with an $[M+H]^+$ of *m/z* 540.3684 and a corresponding labeled ion with an $[M+H]^+$ of *m/z* 548.4186, respectively (Figure 2C). Analysis of tandem MS fragmentation indicated condensation of the amine of Phe with DCA at the terminal side-chain carboxylic acid (Figure S3). Key MS^2 fragments that indicate the position of the phenylalanine on DCA include the doubly dehydroxylated DCA-Phe conjugate ion (*m/z* 504.3472), the *N*-(1-Oxo-4-penten-1-yl)phenylalanine ion (*m/z* 248.1281), the phenylalanine aminium ion (*m/z* 166.0861), and the phenylalanine iminium ion (*m/z* 120.0805). Ions corresponding to the doubly dehydroxylated DCA-Phe conjugate ion and the phenylalanine aminium and iminium ions with eight Dalton increases were observed in MS^2 data associated with an MS^1 scan with an eight Dalton increase in the L-phenylalanine (D_8, 98%) sample, further confirming the structural assignment of phenylalanine conjugation at the

terminal side-chain carboxylic acid of DCA. Additionally, comparison of data from DCA-Phe in our study to that of phenylalanocholic acid (Phe-cholic acid) in Quinn, et al. (2) supports these results. Specifically, we observed matches between the phenylalanine ammonium and iminium ions as well as expected fragment shifts between the two MS^2 spectra arising from the absence of a hydroxyl group in DCA-Phe.

Tyramine conjugate

Comparison of broth from P207 cultures containing DCA and unlabeled tyramine or isotopically labeled tyramine:HCl (1,1,2,2-D_4, 98%) revealed a DCA-tyramine conjugate with an $[M+H]^+$ of *m/z* 512.3740 and a corresponding labeled ion with an $[M+H]^+$ of *m/z* 516.3987, respectively (Figure 2D). Analysis of tandem MS fragmentation indicated condensation of the amine of tyramine with DCA at the terminal side-chain carboxylic acid (Figure S4). Key MS^2 fragments in data from the unlabeled P207 cultures that indicate the position of the tyramine on DCA include the doubly dehydroxylated DCA-tyramine conjugate ion (*m/z* 476.3526), the dehydroxylated DCA-tyramine conjugate ion (*m/z* 494.3629), the *N*-(1-Oxo-4-penten-1-yl)tyramine ion (*m/z* 220.1330), the tyramine cation (*m/z* 138.0913), and the 2-(4-hydroxyphenyl)ethylium ion (*m/z* 121.0644). Ions corresponding to these with four Dalton increases were observed in MS^2 data associated with an MS^1 scan with a four Dalton increase in the tyramine:HCl (1,1,2,2-D_4, 98%) sample, further confirming the structural assignment of tyramine conjugation at the terminal side-chain carboxylic acid of DCA to produce tyraminodeoxycholic acid. The identity of the DCA-tyramine product was further validated by comparing the retention time and MS^2 data of the species with an $[M+H]^+$ of *m/z* 512.3740 to a DCA-tyramine synthetic standard. The matching retention times and MS_2 data confirmed the identity of this molecule as DCA-tyramine (Figure 3 and Figure S5).

GABA conjugate

More specifically, comparison of broth from P207 cultures containing DCA and unlabeled 4-aminobutyric acid (GABA) or isotopically labeled GABA ($^{13}C_4$, 97-99%) revealed a DCA-GABA conjugate with an $[M+H]^+$ of *m/z* 478.3531 and a corresponding labeled ion with an $[M+H]^+$ of *m/z* 482.3666, respectively (Figure 2E). Analysis of tandem MS fragmentation indicated condensation of the primary amine of GABA with DCA at the terminal side-chain carboxylic acid (Figure S5). Key MS^2 fragments in data from the unlabeled P207 cultures that indicate the position of GABA on DCA include the doubly dehydroxylated DCA-GABA conjugate ion (detected *m/z* 442.3316), the *N*-(1-Oxo-4-penten-1-yl)-GABA ion (detected *m/z* 186.1121), the GABA cation (detected *m/z* 104.0706), and the GABA iminium ion (detected *m/z* 86.0596). Samples from GABA ($^{13}C_4$, 97-99%)-labeled broth contained ions with four Dalton increases corresponding to each of these MS^2 fragments, which were associated with an MS^1 scan with a four Dalton increase. These results further support the structural assignment of GABA conjugation at the terminal side-chain carboxylic acid of DCA to produce γ-aminobutyrodeoxycholic acid.

Table S1. Postulated DCA-amine conjugates with predicted positive and negative ions used to search raw mass spectrometry data and molecular networks.

**DCA-AA conjugate**	**$[M+H]^+$**	**$[M-H]^-$**
DCA-Urea	435.3218	433.3072
DCA-Glycine	450.3214	448.3068
DCA-Glycolic acid	451.3054	449.2908
DCA-Alanine	464.3371	462.3225
DCA-beta-Alanine	464.3371	462.3225
DCA-Sarcosine	464.3371	462.3225
DCA-Lactic acid	465.3211	463.3065
DCA-Glycerol	467.3367	465.3221
DCA-Phosphoric acid	473.2663	471.2517
DCA-GABA	478.3527	476.3381
DCA-Serine	480.3320	478.3174
DCA-Uracil	487.3167	485.3021
DCA-ornithine lactam	489.3687	487.3541
DCA-Proline	490.3527	488.3381
DCA-Fumarate	491.3004	489.2858
DCA-Valine	492.3684	490.3538
DCA-Succinic acid	493.3160	491.3014
DCA-Threonine	494.3476	492.3330
DCA-Cysteine	496.3092	494.2946
DCA-Nicotinamide	497.3374	495.3228
DCA-Taurine	500.3041	498.2895
DCA-5-oxoprolinate	504.3320	502.3174
DCA-5-oxoproline	504.3320	502.3174
DCA-L-Isoleucine	506.2894	504.2748
DCA-Leucine	506.3840	504.3694
DCA-Isoleucine	506.3840	504.3694
DCA-Asparagine	507.3429	505.3283
DCA-ornithine	507.3793	505.3647
DCA-Aspartic acid	508.3269	506.3123
DCA-Malate	509.3109	507.2963
DCA-Erythronic acid	511.3266	509.3120
DCA-Hypoxanthine	511.3279	509.3133
DCA-tyramine	512.3735	510.3589
DCA-2-Oxoglutarate	521.3109	519.2963
DCA-Glutamine	521.3585	519.3439
DCA-Lysine	521.3949	519.3803
DCA-Glutamic acid	522.3426	520.3280
DCA-Methionine	524.3405	522.3259
DCA-histidine	530.3589	528.3443
DCA-Phenylalanine	540.3684	538.3538
DCA-selenocysteine	543.2458	541.2312

DCA-Capric acid	547.4357	545.4211
DCA-argninine	549.4011	547.3865
DCA-N-Acetylaspartic acid	550.3375	548.3229
DCA-Tyrosine	556.3633	554.3487
DCA-Azelaic acid	563.3943	561.3797
DCA-N-Acetylglutamic acid	564.3531	562.3385
DCA-Citrate	567.3164	565.3018
DCA-Isocitrate	567.3164	565.3018
DCA-tryptophan	579.3793	577.3647
DCA-Pantothenic acid	594.3994	592.3848
DCA-Myristic acid	603.4983	601.4837
DCA-Palmitoleic acid	629.5140	627.4994
DCA-pyrolysine	630.4477	628.4331
DCA-Palmitic acid	631.5296	629.5150
DCA-Oleic acid	657.5453	655.5307
DCA-Stearic acid	659.5609	657.5463
DCA-Docosahexaenoic acid	703.5296	701.5150
DCA-Cholesterol	761.6443	759.6297
DCA-(2R)-Pyrrolidine	762.9894	760.9748

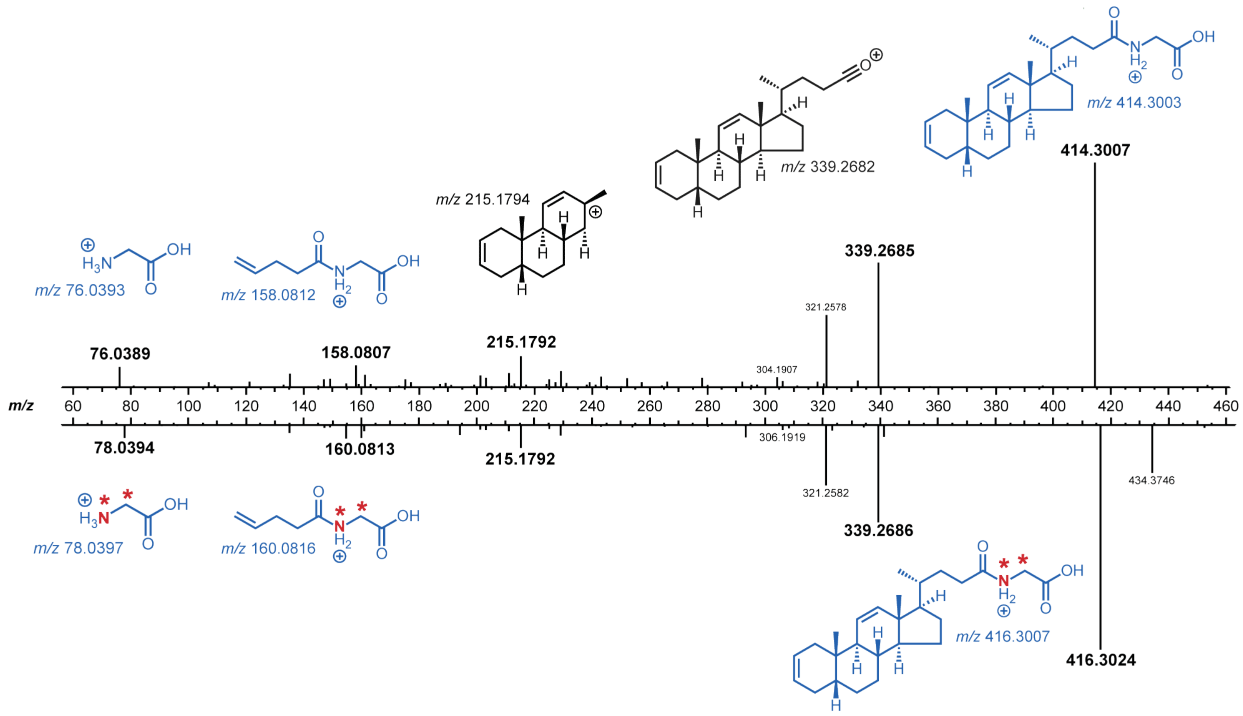

Figure S1. Mirror plot of a summed DCA-Gly (*m/z* 450.3220) MS^2 spectrum opposite a summed MS^2 spectrum for the isotopically labeled DCA-^{15}N,^{13}C-Gly (*m/z* 452.3230), which was observed only when *B. fragilis* P207 was cultured in the presence of DCA and ^{15}N,^{13}C-Gly (Figure 2). The +2 deuterium shift was observed in the expected MS^2 fragments between the spectra. (red asterisk indicating location of deuteriums). All structural assignments for MS2 ions are putative.

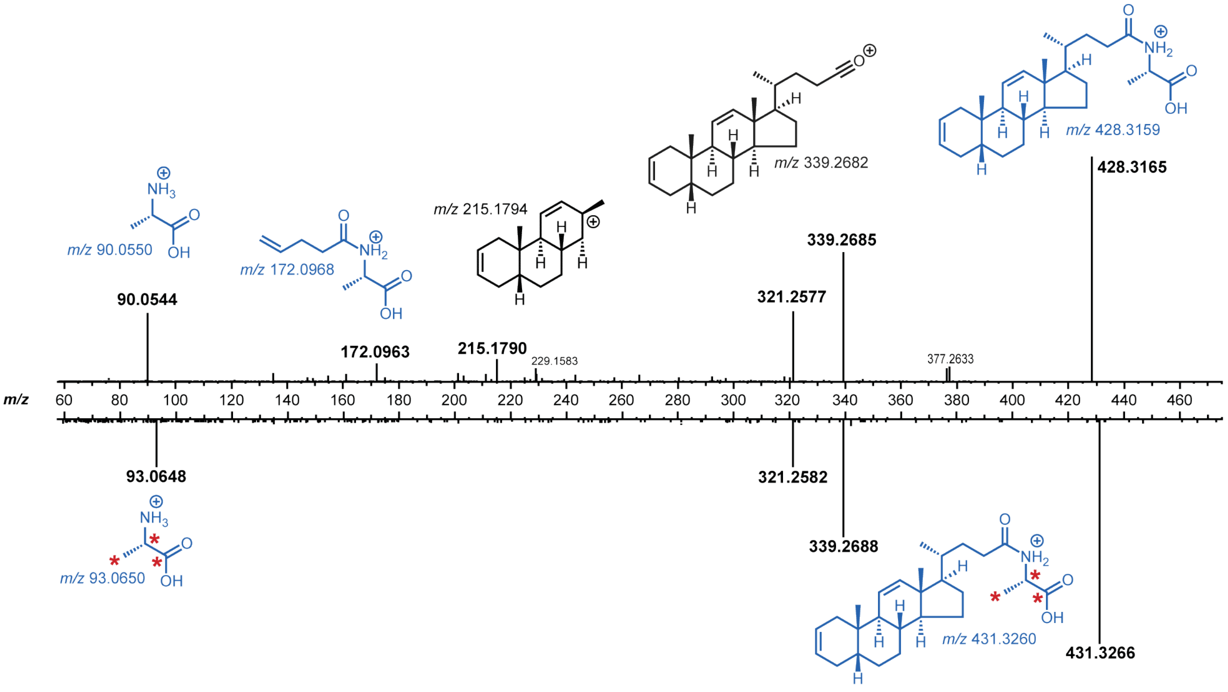

Figure S2. Mirror plot of a summed DCA-Ala (*m/z* 464.3373) MS^2 spectrum opposite a summed MS^2 spectrum for the isotopically labeled DCA-$^{13}C_3$-Ala (*m/z* 467.3475), which was observed only when *B. fragilis* P207 was cultured in the presence of DCA and $^{13}C_3$-Ala (Figure 2). The +3 deuterium shift was observed in the expected MS^2 fragments between the spectra (red asterisk indicating location of deuteriums). All structural assignments for MS2 ions are putative.

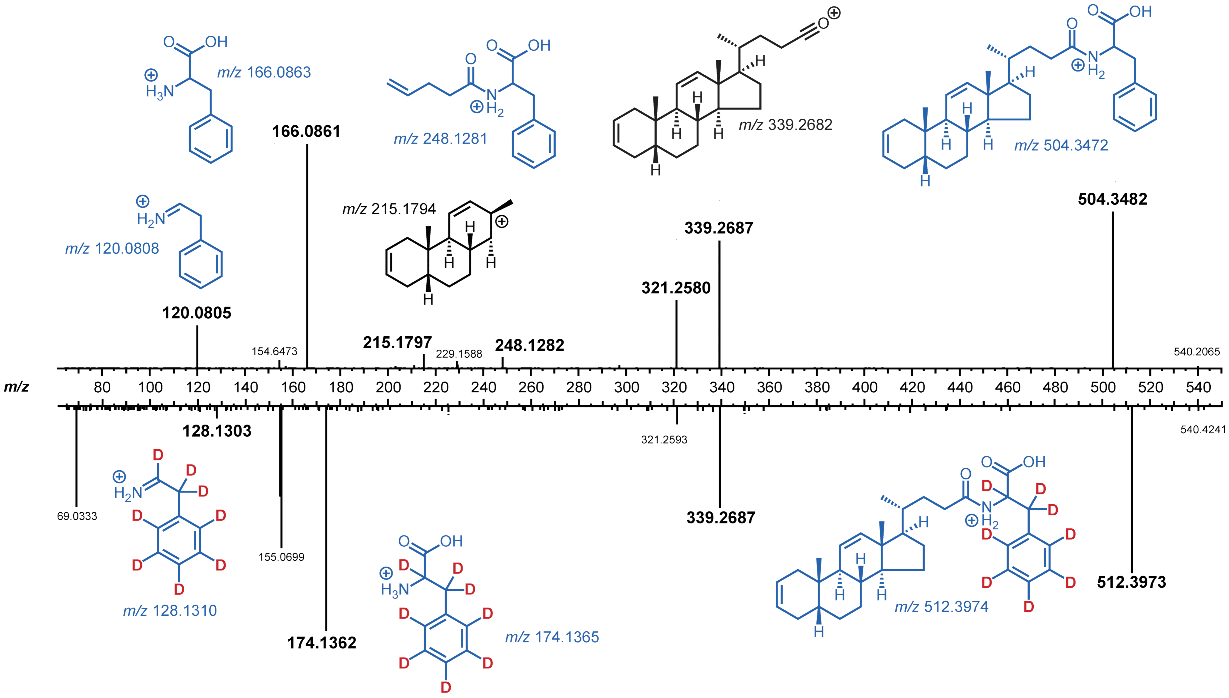

Figure S3. Mirror plot of a summed DCA-Phe (*m/z* 540.3686) MS^2 spectrum opposite a summed MS^2 spectrum for the isotopically labeled DCA-D_8-Phe (*m/z* 548.4186), which was observed only when *B. fragilis* P207 was cultured in the presence of DCA and D_8-Phe (Figure 2). The +8 deuterium shift was observed in the expected MS^2 fragments between the spectra (red text indicating location of deuteriums). All structural assignments for MS2 ions are putative.

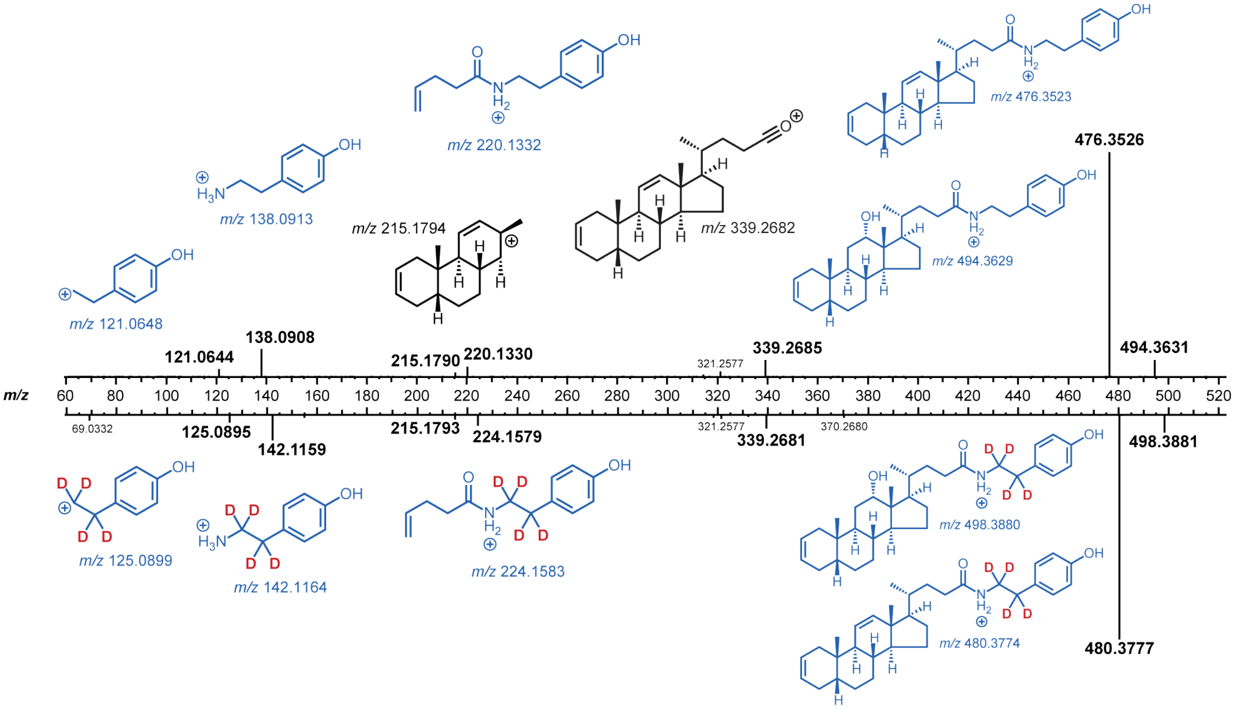

Figure S4. Mirror plot of a summed DCA-tyramine (*m/z* 512.3740) MS^2 spectrum opposite a summed MS^2 spectrum for the isotopically labeled DCA-D_4-tyramine (*m/z* 516.3987), which was observed only when *B. fragilis* P207 was cultured in the presence of DCA and D_4-tyramine (Figure 2). The +4 deuterium shift was observed in the expected MS^2 fragments between the spectra (red asterisk indicating location of deuteriums). All structural assignments for MS2 ions are putative.

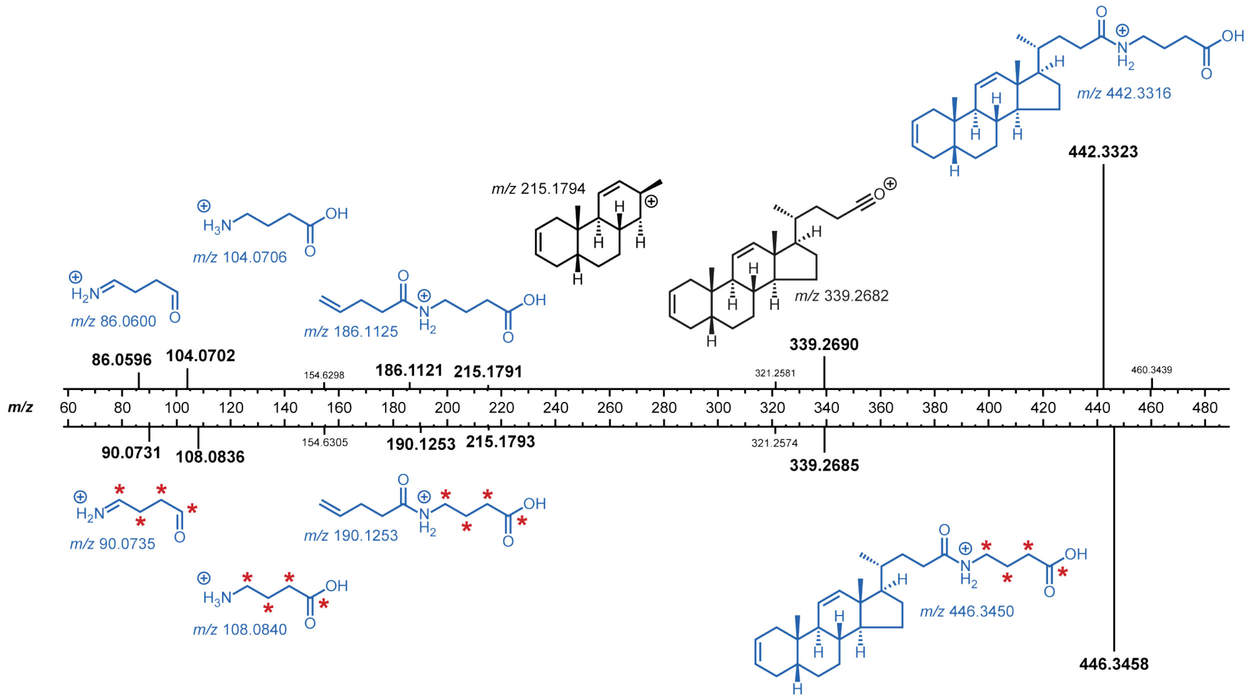

Figure S5. Mirror plot of a summed DCA-GABA (*m/z* 478.3531) MS^2 spectrum opposite a summed MS^2 spectrum for the isotopically labeled DCA-$^{13}C_4$-GABA (*m/z* 482.3666), which was observed only when *B. fragilis* P207 was cultured in the presence of DCA and $^{13}C_4$-GABA (Figure 2). The +4 deuterium shift was observed in the expected MS^2 fragments between the spectra (red asterisk indicating location of deuteriums). All structural assignments for MS2 ions are putative.

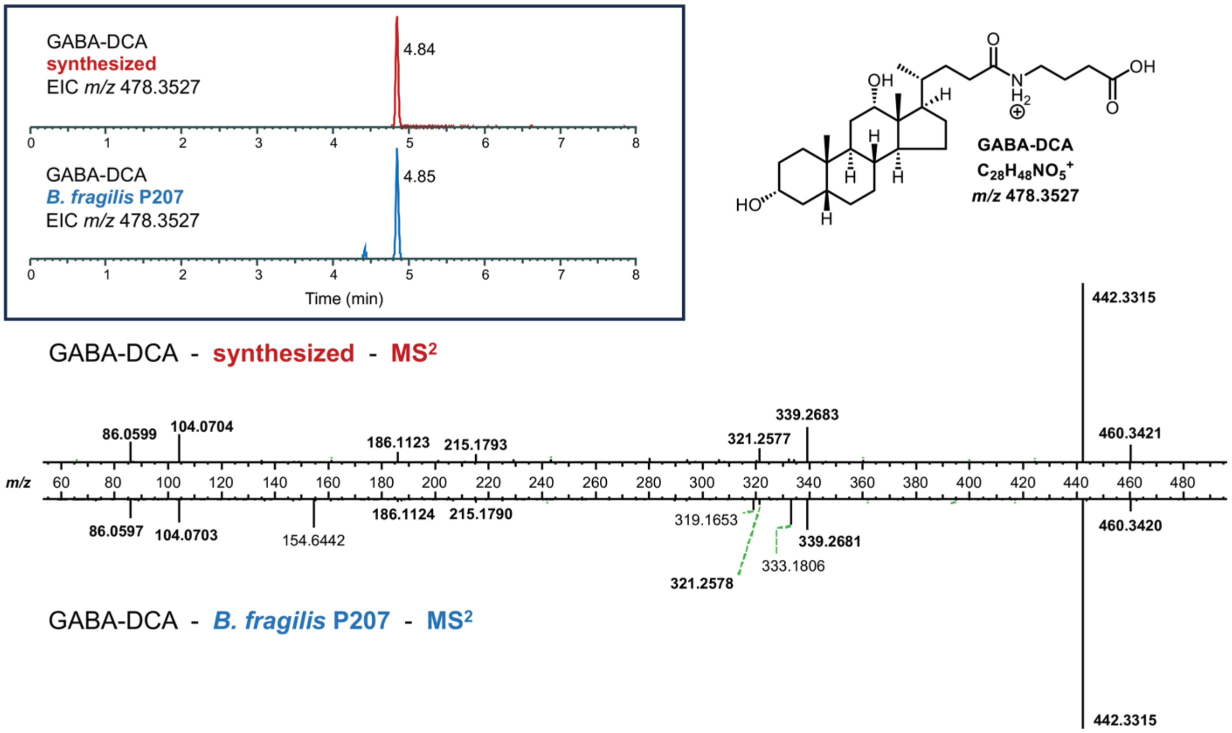

Figure S6. Comparative LC-MS/MS analysis of DCA-GABA produced by *B. fragilis* P207 *in vitro* to a synthetic standard. (Top left) Extracted ion chromatogram (EIC) of chemically synthesized DCA-GABA (m/z 478.3527) (retention time=4.84 minutes) and the EIC for DCA-GABA identified in *B. fragilis* P207 broth cultures (bottom; retention time=4.85 minutes). (bottom) Mirror plot of a summed DCA-GABA MS^2 spectrum for the synthetic standard opposite a summed MS^2 spectrum for the DCA-GABA detected in *B. fragilis* P207 broth cultures.

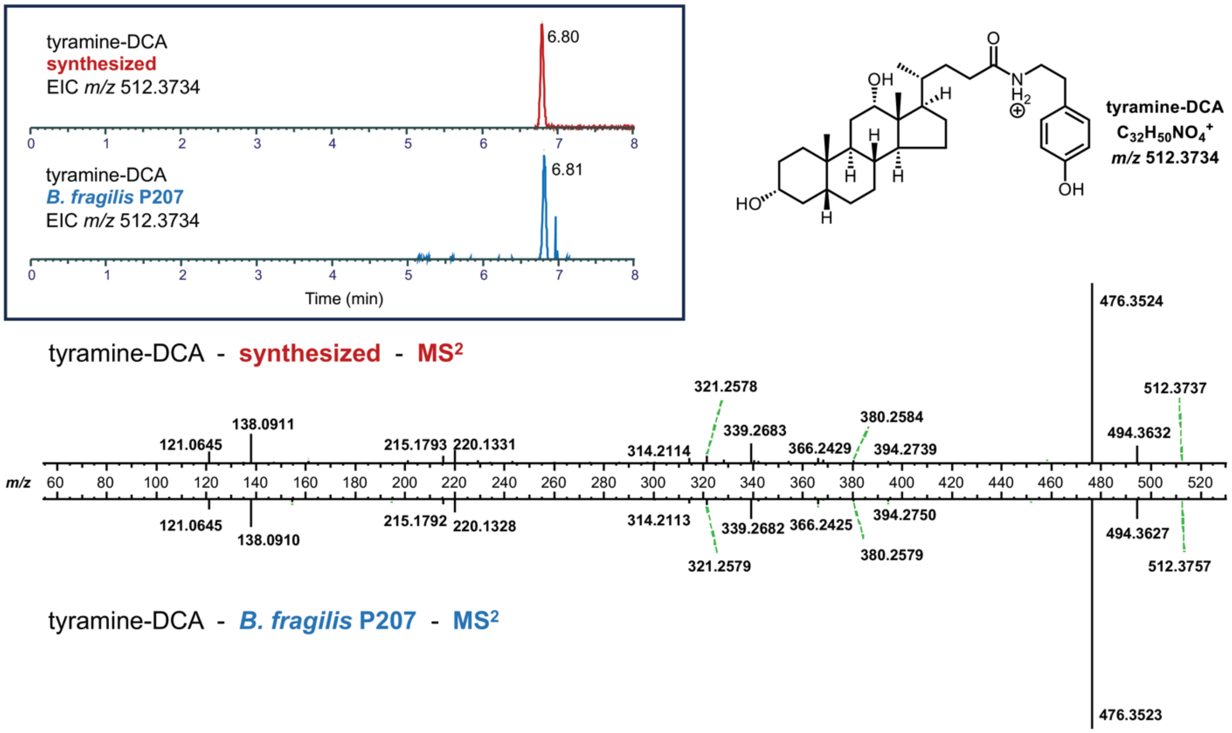

Figure S7. Comparative LC-MS/MS analysis of DCA-tyramine produced by *B. fragilis* P207 *in vitro* to a synthetic standard. (Top left) Extracted ion chromatogram (EIC) of chemically synthesized DCA-tyramine (m/z 512.3734) (retention time=6.80 minutes) and the EIC for DCA-tyramine identified in *B. fragilis* P207 broth cultures (bottom; retention time=6.81 minutes). (bottom) Mirror plot of a summed DCA-GABA MS^2 spectrum for the synthetic standard opposite a summed MS^2 spectrum for the DCA-GABA detected in *B. fragilis* P207 broth cultures.

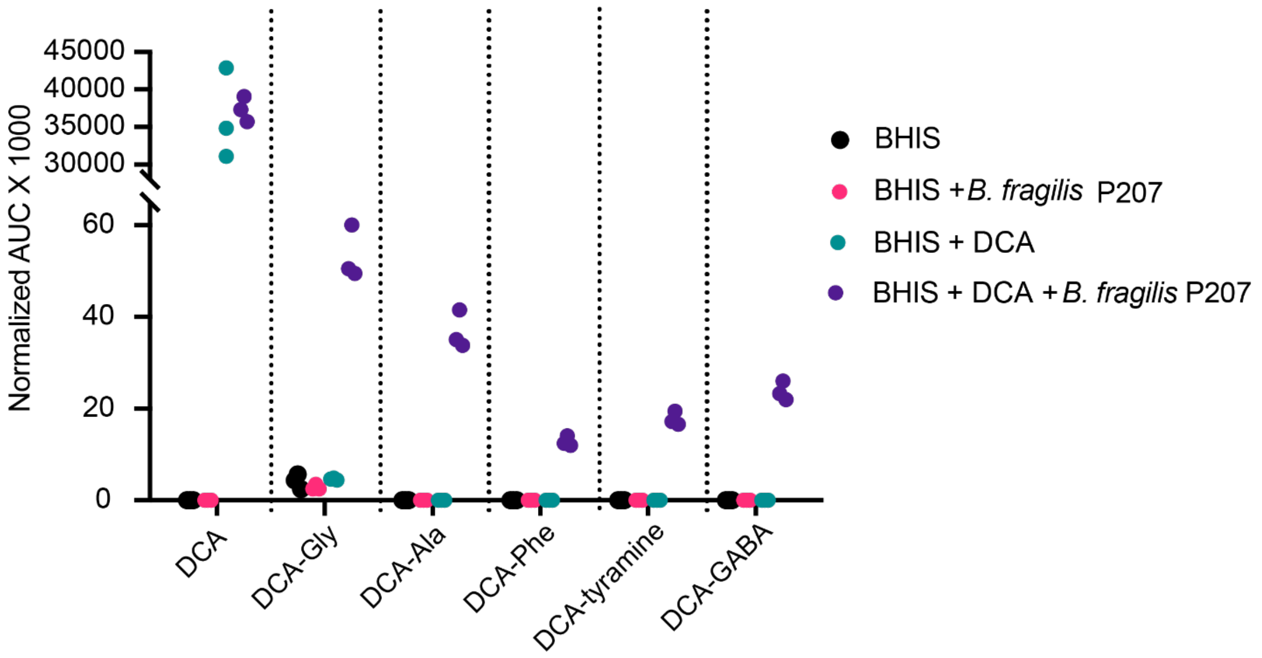

Figure S8. Relative abundances of the five deoxycholic acid (DCA)-amine conjugates in triplicate samples of BHIS media, BHIS media with *B. fragilis* strain P207 (P207), BHIS media with DCA, and BHIS media with P207 and DCA normalized to the average peak area under the curve (AUC) for internal standard ions D_4-taurocholic acid $[M+H]^+$ at *m/z* 520.3241, D_4-taurodeoxycholic acid $[M+H]^+$ at *m/z* 504.3291, and D_4-glycocholic acid at m/z 470.3414.

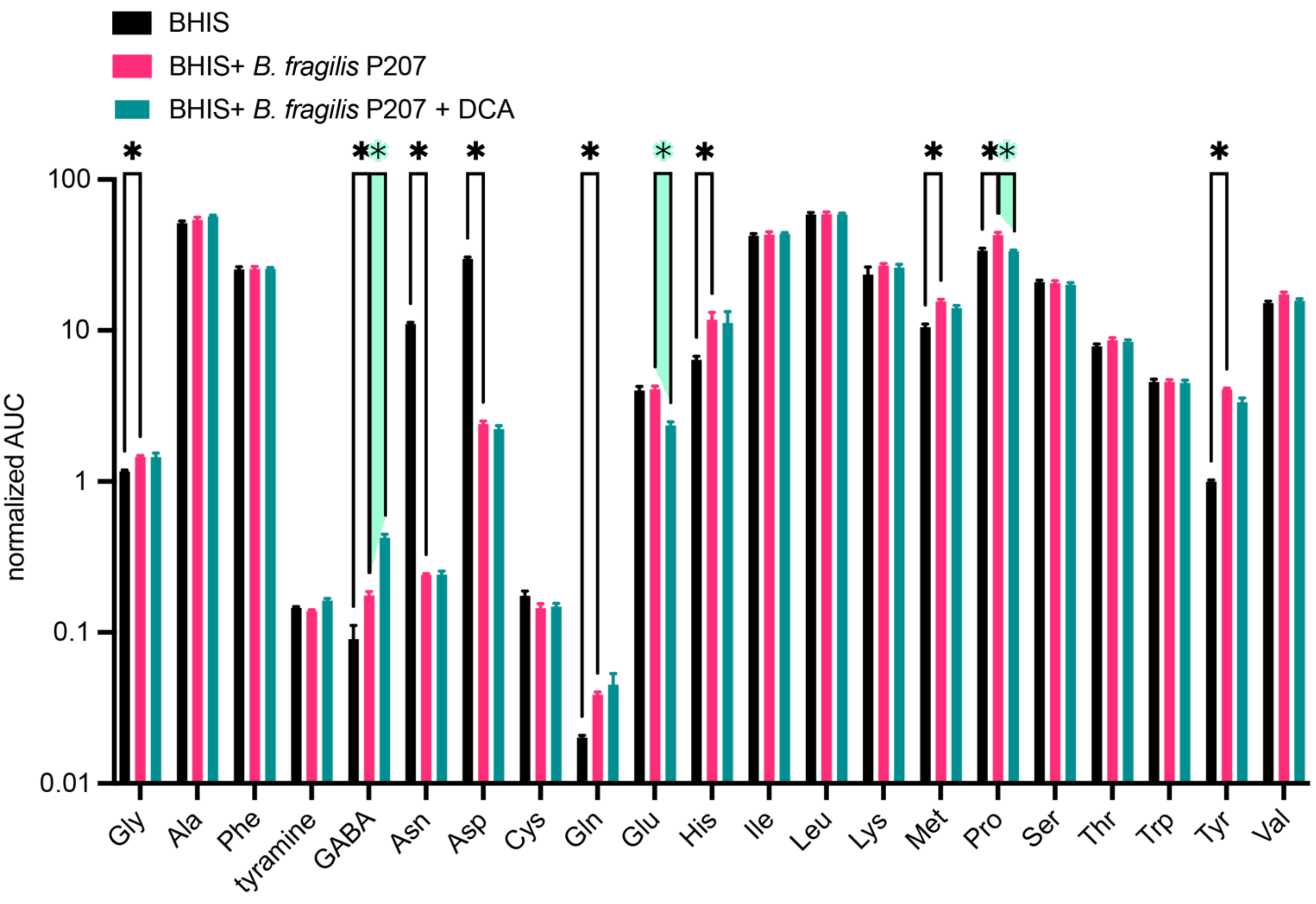

Figure S9. Comparative GC/MS-based quantification of select amines in *B. fragilis* P207 cultures. The cultures were incubated in BHIS medium either without (blue bars) or with (pink bars) the addition of deoxycholic acid (DCA) at a concentration of 0.01% (w/v). BHIS medium alone is shown in black bars. The graph presents the relative concentrations, derived from the area under the curve (AUC) of detected peaks, indicating the comparative levels of these amines across the different conditions. Each bar denotes the mean of three independent experiments, and error bars represent the standard deviations. Statistical significance between conditions was assessed using individual t-tests, with corrections for multiple comparisons via the two-stage step-up method of Benjamini, Krieger, and Yekutieli; significance is indicated by an asterisk (*), denoting a q-value of less than 0.01.

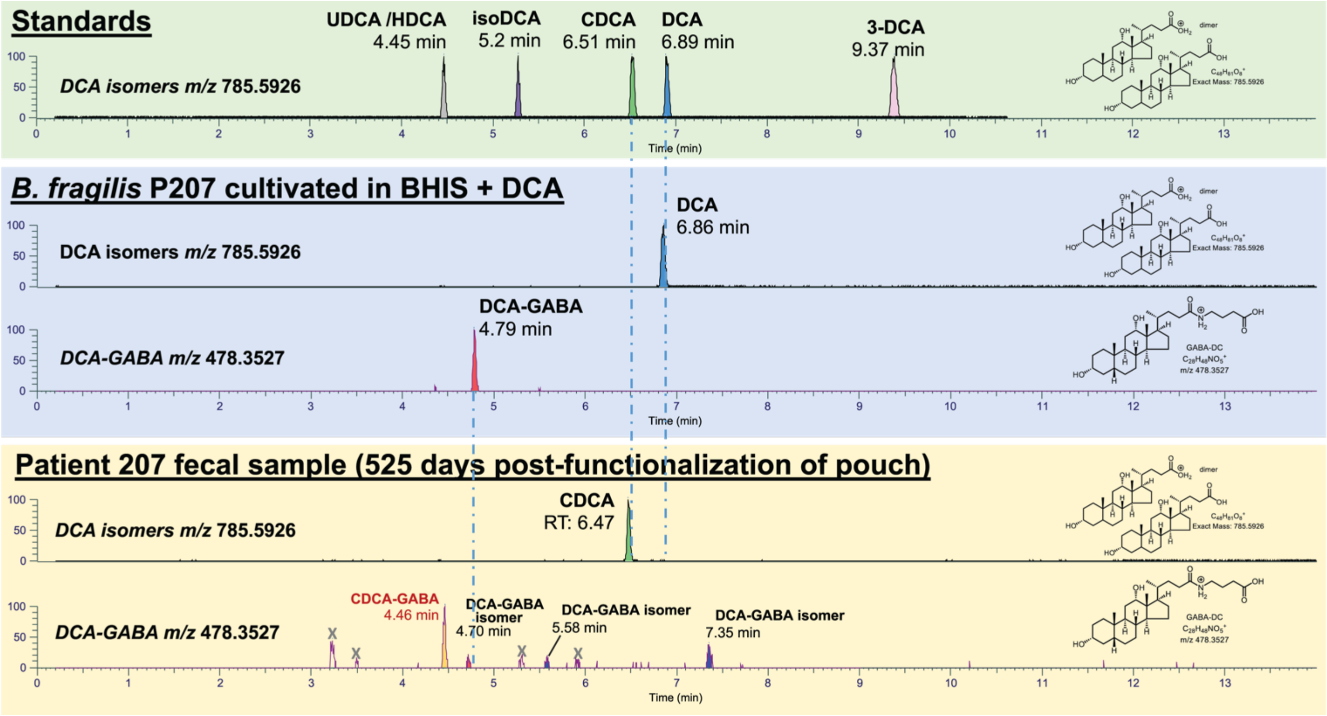

Figure S10. (Top panel) Liquid chromatography temporal elution profile of deoxycholic acid (DCA) and DCA isomer standards (*m/z* dimer 785.5926): ursodeoxycholic acid (UDCA); hyodeoxycholic acid (HDCA); chenodeoxycholic acid (CDCA). (Middle panel) Extracted ion chromatogram of resolved BHIS media containing *B. fragilis* strain P207 with 0.01% (w/v) DCA; *m/z* for DCA (dimer) and DCA-GABA. Quantification of all five DCA conjugates detected in *B. fragilis* strain P207 culture extract by MS/MS are presented in Figure S8 and Table S2. (bottom panel) Extracted ion chromatogram of fecal extract of human pouchitis patient 207 at 525 days post pouch functionalization; *m/z* for DCA (dimer) and DCA-GABA. This analysis was conducted on multiple fecal collection time points and yielded multiple peaks with MS/MS profiles matching GABA conjugated to DCA. The absence of a clear peak at Rt ≈4.8 minutes (corresponding to the DCA-GABA standard; see Figure 3), and the lack of DCA in patient 207 stool (Rt ≈ 6.9) suggests these species are DCA isomer-GABA conjugates (labeled DCA-GABA isomer). LC-MS/MS analysis of a synthetic GABA-CDCA standard showed that the major GABA conjugate peak at this *m/z* is CDCA-GABA. Quantification of bile acid peaks in longitudinal fecal samples across an approximate 2-year period after surgical functionalization of the ileal pouch is presented in Table S2.

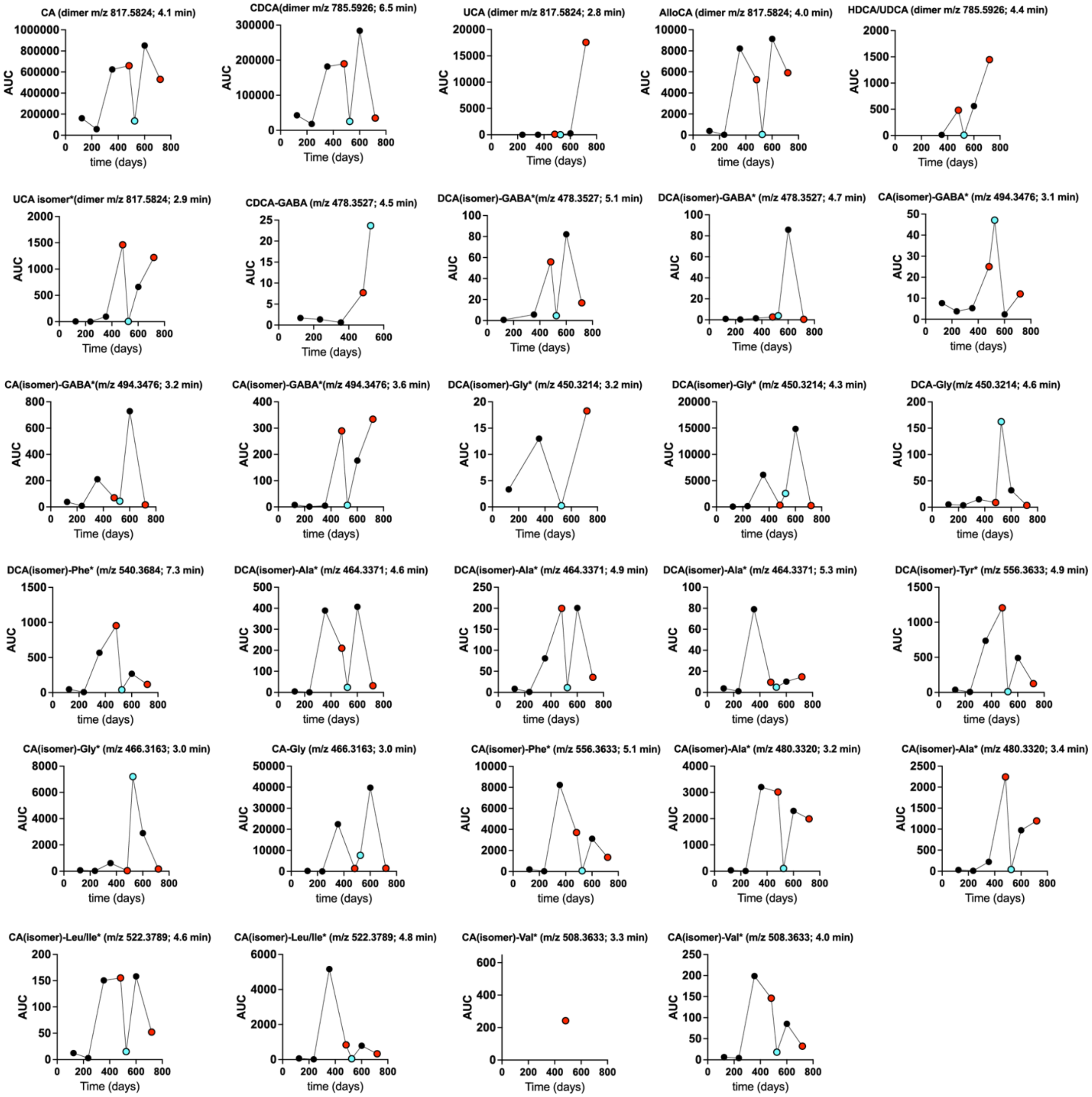

Figure S11. Longitudinal profile of unconjugated and conjugated bile acid species in pouchitis patient 207 from 124 to 719 days post-functionalization of the ileal J-pouch (3). Red dots at 482 and 719 days post-functionalization are clinic visits where the patient was diagnosed with pouch inflammation (i.e. pouchitis). Blue dot (525 days) was during a period of antimicrobial treatment (ciprofloxacin), in which the patient did not have pouchitis. The top of each graph notes the elution time of each species in minutes. Synthetic standards of hyodeoxycholate/ursodeoxycholate (HDCA/UDCA), chenodeoxycholate (CDCA), cholic acid (CA), deoxycholic acid (DCA), 3-CDA, iso-DCA, ursocholic acid (UCA), and litchcholic acid (LCA) were included in this analysis. DCA (3α,12α-Dihydroxy-5β-cholan-24-oic acid) was absent at all time points in this patient, so we expect the labeled DCA(isomer) amide conjugates have a CDCA core, though this is not proven in most cases. Likewise, we expect that many of the abundant CA(isomer) conjugates have a cholic acid core though the particular isomer/epimer of these products has not been defined. Points are missing for compounds at timepoints where they were not detected.

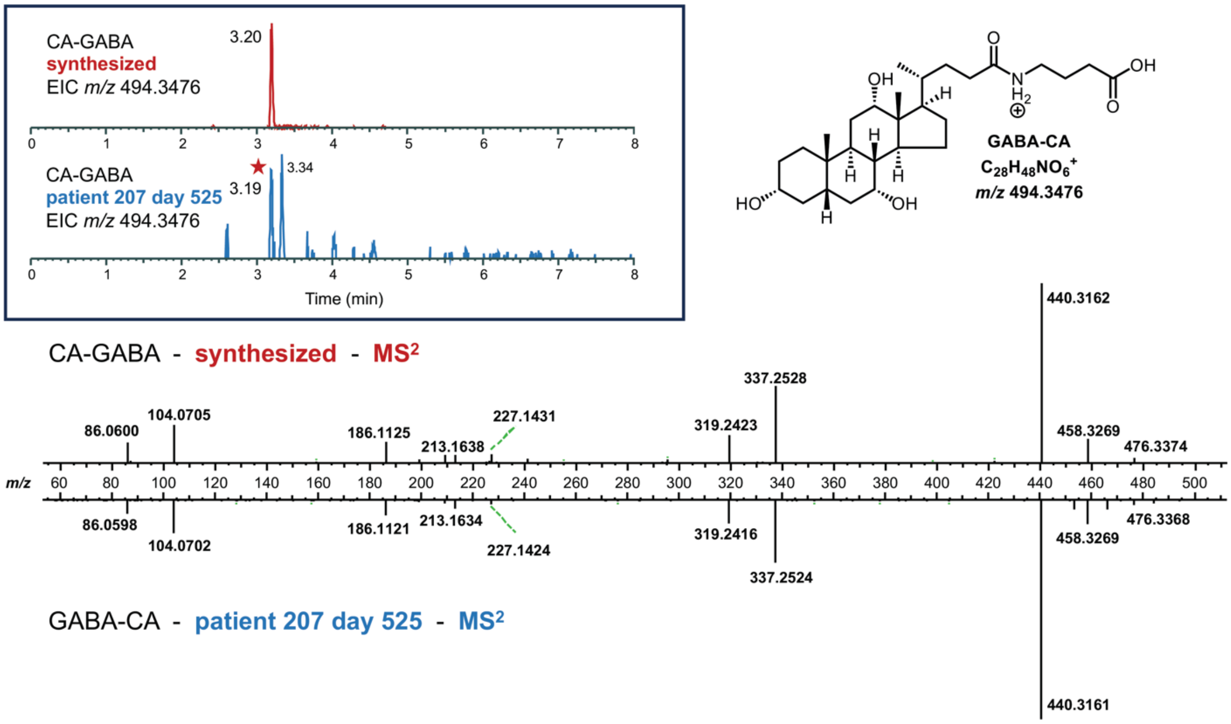

Figure S12. Comparative LC-MS/MS analysis of CA-GABA in patient 207 stool to a synthetic standard. (Top left) Extracted ion chromatogram (EIC) of chemically synthesized CA-GABA (*m/z* 494.3476) (retention time=3.20 minutes) and the EIC for CA-GABA identified in patient 207 stool at 525 days post-functionalization of the ileal pouch (bottom; retention time=3.19 minutes). (bottom) Mirror plot of a summed CA-GABA MS^2 spectrum for the synthetic standard opposite a summed MS^2 spectrum for the CA-GABA detected in patient 207 stool.

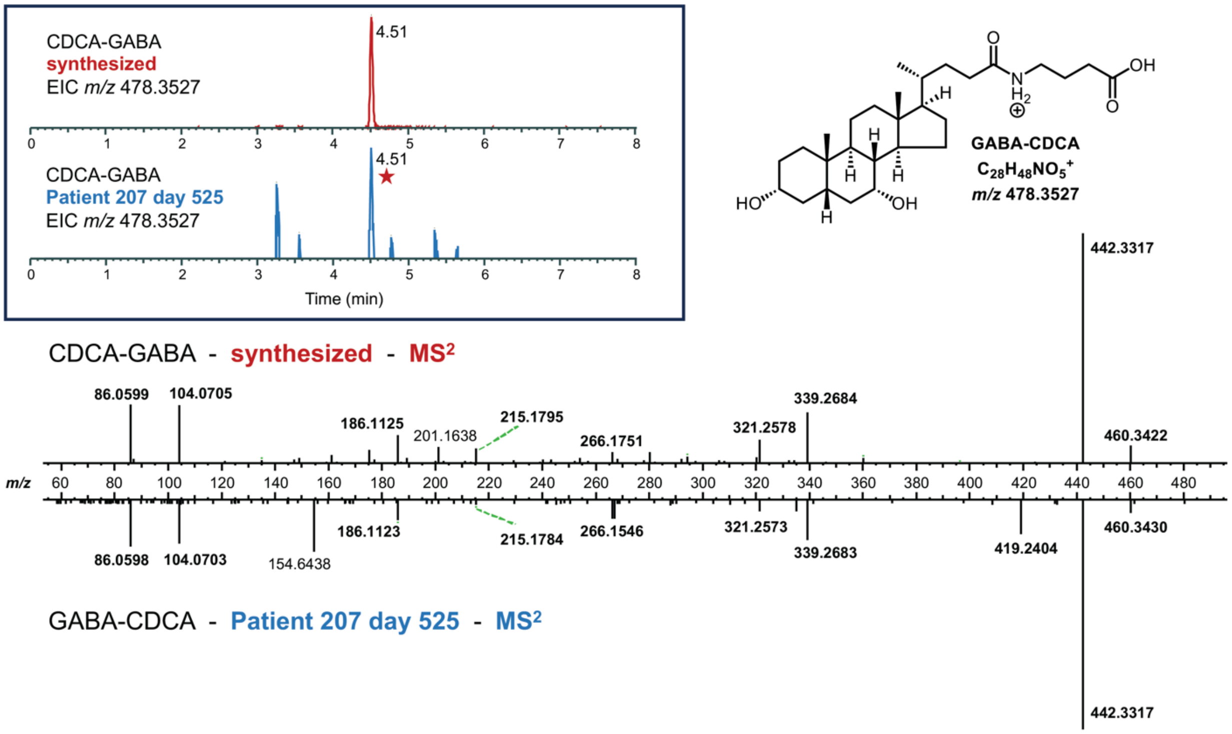

Figure S13. Comparative LC-MS/MS analysis of CDCA-GABA in patient 207 stool to a synthetic standard. (Top left) Extracted ion chromatogram (EIC) of chemically synthesized CDCA-GABA (*m/z* 478.3527) (retention time=4.51 minutes) and the EIC for CDCA-GABA identified in patient 207 stool at 525 days post-functionalization of the ileal pouch (bottom; retention time=4.51 minutes). (bottom) Mirror plot of a summed CDCA-GABA MS^2 spectrum for the synthetic standard opposite a summed MS^2 spectrum for the CDCA-GABA detected in patient 207 stool.

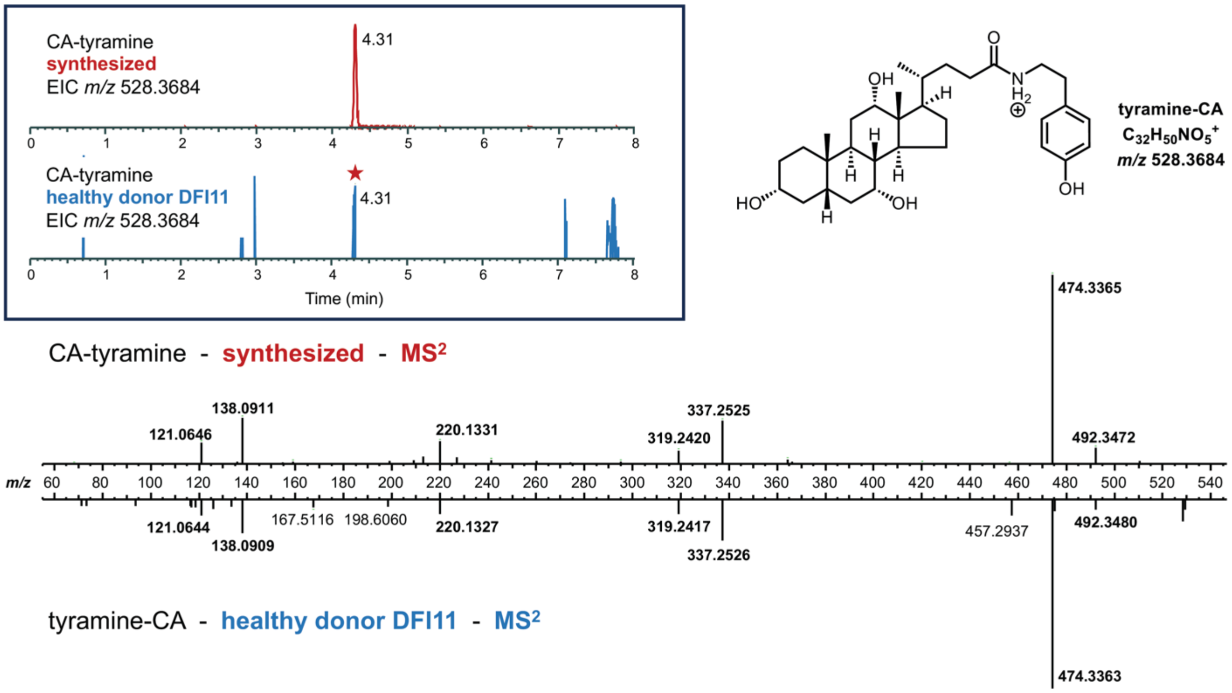

Figure S14. Comparative LC-MS/MS analysis of CA-tyramine in healthy human donor (DFI11) stool to a synthetic standard. (Top left) Extracted ion chromatogram (EIC) of chemically synthesized CA-tyramine (*m/z* 528.3684) (retention time=4.31 minutes) and the EIC for CA-tyramine identified in healthy donor DFI11 stool (bottom; retention time=4.31 minutes). (bottom) Mirror plot of a summed CA-tyramine MS^2 spectrum for the synthetic standard opposite a summed MS^2 spectrum for the CA-tyramine in DFI11 donor stool.

```
                                        cov    pid     1 [                                                                              ] 80
1 B. fragilis WP_005817456.1   100.0% 100.0%   ------MATLIVIAVNFIWSGQPVKACTRAVYIGPDNMVITGRTMDWKEDIQ-SNLYLFPRGIKRAGYNKG--NTVEWIS
2 B. ovatus   WP_217723859.1   100.0%  79.0%   MKKK-LAGVALVLAVVSLMSIQPVEACTRAVYIGPDQMVVTGRTMDWKEDIM-TNIYVFPRGIQRMGHNKE--KTVNWTS
3 B. ovatus   WP_004308262.1    94.2%  53.7%   -------------------------MCTRVVYSGKNGMVVTGRSMDWKTEMH-SNLWVFPKGMERNGETGA--NSLQWTS
4 B. ovatus   WP_004323538.1    91.6%  22.1%   MTKNLLLGIA-----AVCGSTFQAVACTGISLTSRDGSYVQARTIEWARGVLQSEYVIIPRGQQLTSFTPTGVNGLTFTA
5 M. gnavus   WP_004614568.1    86.4%  18.8%   -------------------------MCTAATYK--TKDFYFGRTLDYEFSYG-DQIVITPRNYSFHFR-HVG--D--MEH
6 B. longum   WP_007052221.1    83.8%  19.7%   -------------------------MCTGVRFSDDEGNTYFGRNLDWSFSYG-ETILVTPRGYHYDTVFGAG--G--KAK

                                        cov    pid    81                                                                                160
1 B. fragilis   WP_005817456.1 100.0% 100.0%   KYGSIVAT---GYDIGTCDGMNEKGLVASLLFLPESI-YVR--PNDTRPVMGISIWTQYVLDNFATVSEAVEELKKQTFR
2 B. ovatus     WP_217723859.1 100.0%  79.0%   KYGSVIAT---GYDIGTCDGMNEKGLVASLLFLPESI-YSL--PGDTRPAMGISIWTQYVLDNFATVREAVDELKKETFR
3 B. ovatus     WP_004308262.1  94.2%  53.7%   KYGSVVTS---AFEIASTDGMNEKGLVANLLWLPETE-YPV--RDQSKPGLAITAWVQYMLDNFATVDEAVAFIDENTFQ
4 B. ovatus     WP_004323538.1  91.6%  22.1%   KYGVVGLAVVQK--EFIAEGINEAGLSAGLFFFPHYGGYETYDAAQNQRTLADLQVTEWLLSQFSTIDEVKAALSS--VR
5 M. gnavus     WP_004614568.1  86.4%  18.8%   HYAIIGMAHVAEDYPLYYDAMNEKGVAMAGLNFVGNAAYSE--VQSNVENIAQFEFISWILSQCASLVEVRELLDR--IN
6 B. longum     WP_007052221.1  83.8%  19.7%   PNAVIGVGVVMADRPMYFDCANEHGLAIAGLNFPGYASFVH-EPVEGTENVATFEFPLWVARNFDSVDEVEEALRN--VT

                                        cov    pid   161                                                                                240
1 B. fragilis   WP_005817456.1 100.0% 100.0%   IDAPDMPNGS-ASTLHMAITDETGNSAVLEYIDGNLIIHEGKEYQVMTNSPRYDLQLAV-NDY-----------------
2 B. ovatus     WP_217723859.1 100.0%  79.0%   IDAPRMPNGGPESTLHLAITDETGNTAVLEYLDGKLSIHEGKEYRVMTNSPRYEQQLAI-NDY-----------------
3 B. ovatus     WP_004308262.1  94.2%  53.7%   VVSDLMPDGSRLATLHLSISDATGDCAIFEYTGGKLTVYHSKEYKVMTNSPTYNKQLAL-NEY-----------------
4 B. ovatus     WP_004323538.1  91.6%  22.1%   VVG-----LEKTAVVHWRIGEPSGRQVVLEIVGGVPHFYEN-TVGVLTNAPGFEWQLTNLNNYVNLHPGDASMQKLSGIT
5 M. gnavus     WP_004614568.1  86.4%  18.8%   IVNTPFSEQLPLAQLHWIISDENES-ITVESMSDGLHIYDN-PVGVLTNNPPFPQQMFQLNNYMYLSPKQPENTFCENLA
6 B. longum     WP_007052221.1  83.8%  19.7%   LVSQIVPG-QQESLLHWFIGDGKRS-IVVEQMADGMHVHHD-DVDVLTNQPTFDFHMENLRNYMCVSNEMAEPTSWGKAS

                                        cov    pid   241                                                                                320
1 B. fragilis   WP_005817456.1 100.0% 100.0%   ---WKEVGGLNMLPGTNRSSDRFVRASFYIHAIPQTSDAKIAVPSVLSVMRNVSVPFGITTPDKPYI---SSTRWRSVSD
2 B. ovatus     WP_217723859.1 100.0%  79.0%   ---WKEIGGLQMLPGTNRASDRFVRASFYIHAIPQIADAKIAVPSVLSVMRNVSVPFGINTPEKPYI---SSTRWRSVSD
3 B. ovatus     WP_004308262.1  94.2%  53.7%   ---WKSIGGLSFLPGTNRPSDRFARASFYINALPQTDDVRIAIASVFSVIRNTSVPYGISTPEFPEI---STTQWRTVSD
4 B. ovatus     WP_004323538.1  91.6%  22.1%   LQPTGGNSGFLGIPGDATPPSRFVRAAFYRGTAPQRATGFDTVQQCFHLLNNFDIPIGIEHSQGDIPDIPSATQWTSAID
5 M. gnavus     WP_004614568.1  86.4%  18.8%   LDAYSRGMGGLGLPGDLSSSSRFVRVAFTKVNAISGESEAESVSQFFHILGSVDQQRGCCEVADGKC---EITLYTSCCN
6 B. longum     WP_007052221.1  83.8%  19.7%   LTAWGAGVGMHGIPGDVSSPSRFVRVAYTNAHYPQQNDEAANVSRLFHTLGSVQMVDGMAKMGDGQF---ERTLFTSGYS

                                        cov    pid   321                                                                ] 386
1 B. fragilis   WP_005817456.1 100.0% 100.0%   QKNRVYYFESTLTPNLFWIDLHKVDFSPNASIKKLSLTHGEVYAGDVVKDFKDSRSFTFMFELPQ-
2 B. ovatus     WP_217723859.1 100.0%  79.0%   QKNKVYYFESTLTPNLFWLDLKKIDFSPKAGIKKLSLTKGEIYAGDAVKDLKDSQSFTFLFETPVI
3 B. ovatus     WP_004308262.1  94.2%  53.7%   SKNLLYFFESSLTPNTFWVNLRETDLSEGAPVLKLSIANGETYHGNATKEFKPAQPFRFMGVKG--
4 B. ovatus     WP_004323538.1  91.6%  22.1%   LTNRKVYYKTAYNNSIRCIDLKAIDFSKVKYQSHPLDKI----QEQPVEMVKIPR-----------
5 M. gnavus     WP_004614568.1  86.4%  18.8%   VAKGIYYYNTYENHQISAVDMHVENLDNNKLICYPVIQ------GERINYQNK-------------
6 B. longum     WP_007052221.1  83.8%  19.7%   SKTNTYYMNTYDDPAIRSYAMADYDMDSSELISVAR------------------------------
```

Figure S15. Multiple sequence alignment of N-terminal nucleophilic cysteine hydrolase (Ntn) enzymes (Conserved Domain Database accession cd01902) from *B. fragilis* P207 (reference sequence for this alignment; WP_005817456.1), *Mediterraneibacter gnavus* MSK15.77 (WP_004614568.1), *Bifidobacterium longum* DFI.2.45 (WP_007052221.1), and *Bacteroides ovatus* MSK22.29 (which encodes three Ntn paralogs: WP_217723859.1, WP_004308262.1, and WP_004323538.1). cov-percent coverage; pid-percent identity.

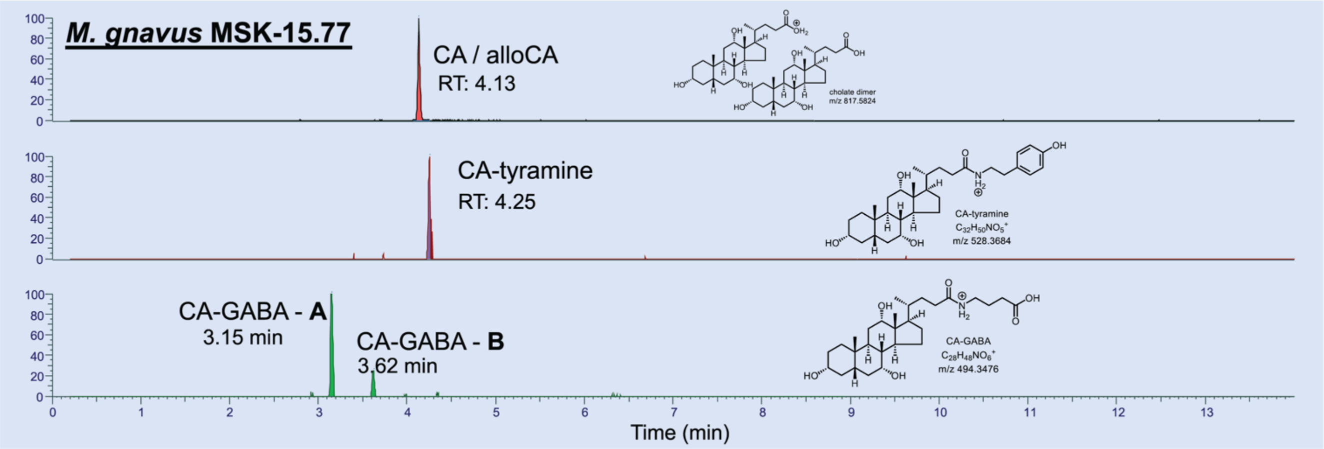

Figure S16. Incubation of *M. gnavus* strain MSK-15.77 with 0.01% (w/v) cholic acid (CA) in BHIS broth followed by separation of culture extract by liquid chromatography provides evidence for production of CA-tyramine and two species with MS/MS profiles matching CA-GABA (putative isomers).

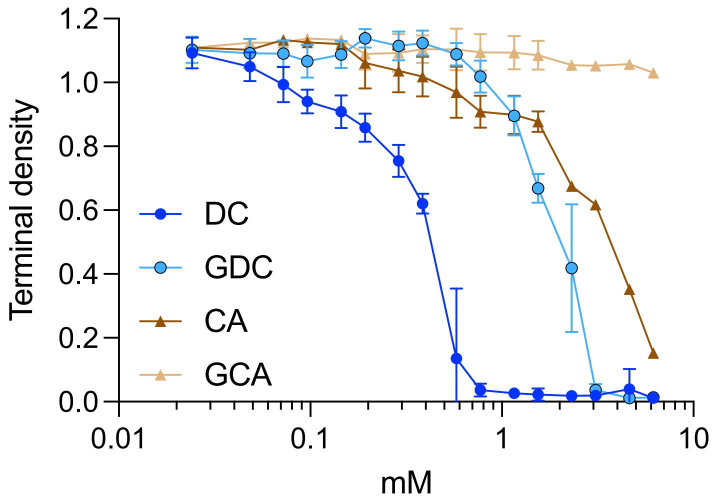

Figure S17. Glycine conjugation to DCA and CA reduces toxicity to *B. fragilis* P207 *in vitro*. Terminal density measurements (OD_{600}) of *B. fragilis* P207 (24 hours of growth) in BHIS under increasing concentrations of bile acids (DC-deoxycholic acid; GDC-glycodeoxycholic acid; CA-cholic acid; GCA-glycocholic acid). Data show five biological replicates for DC and CA, and three replicates for GDC and GCA; error bars represent standard deviation.

References

1. Zhu QF, Wang YZ, An N, Hao JD, Mei PC, Bai YL, Hu YN, Bai PR, Feng YQ. 2022. Alternating Dual-Collision Energy Scanning Mass Spectrometry Approach: Discovery of Novel Microbial Bile-Acid Conjugates. Anal Chem 94:2655-2664. doi: 10.1021/acs.analchem.1c05272.
2. Quinn RA, Melnik AV, Vrbanac A, Fu T, Patras KA, Christy MP, Bodai Z, Belda-Ferre P, Tripathi A, Chung LK, Downes M, Welch RD, Quinn M, Humphrey G, Panitchpakdi M, Weldon KC, Aksenov A, da Silva R, Avila-Pacheco J, Clish C, Bae S, Mallick H, Franzosa EA, Lloyd-Price J, Bussell R, Thron T, Nelson AT, Wang M, Leszczynski E, Vargas F, Gauglitz JM, Meehan MJ, Gentry E, Arthur TD, Komor AC, Poulsen O, Boland BS, Chang JT, Sandborn WJ, Lim M, Garg N, Lumeng JC, Xavier RJ, Kazmierczak BI, Jain R, Egan M, Rhee KE, Ferguson D, Raffatellu M, Vlamakis H, et al. 2020. Global chemical effects of the microbiome include new bile-acid conjugations. Nature 579:123-129. doi: 10.1038/s41586-020-2047-9.
3. Vineis JH, Ringus DL, Morrison HG, Delmont TO, Dalal S, Raffals LH, Antonopoulos DA, Rubin DT, Eren AM, Chang EB, Sogin ML. 2016. Patient-Specific Bacteroides Genome Variants in Pouchitis. mBio 7. doi: 10.1128/mBio.01713-16.
